# Structural‐Functional Pluralistic Modification of Silk Fibroin via MOF Bridging for Advanced Wound Care

**DOI:** 10.1002/advs.202204553

**Published:** 2022-10-28

**Authors:** Zhou Zhu, Yanhua Liu, Junyu Chen, Zihan He, Pengfei Tan, Yong He, Xibo Pei, Jian Wang, Lin Tan, Qianbing Wan

**Affiliations:** ^1^ State Key Laboratory of Oral Diseases National Clinical Research Center for Oral Diseases West China Hospital of Stomatology Sichuan University Chengdu 610041 China; ^2^ State Key Laboratory of Fluid Power and Mechatronic Systems School of Mechanical Engineering Zhejiang University Hangzhou 310027 China; ^3^ College of Biomass Science & Engineering State Key Laboratory of Polymer Materials Engineering Sichuan University Chengdu 610065 China

**Keywords:** adhesive hydrogel, metal organic frameworks, secondary structure, silk fibroin, wound healing

## Abstract

Silk fibroin (SF) is widely used to fabricate biomaterials for skin related wound caring or monitoring, and its hydrogel state are preferred for their adaptability and easy to use. However, in–depth development of SF hydrogel is restricted by their limited mechanical strength, increased risk of infection, and inability to accelerate tissue healing. Therefore, a structure–function pluralistic modification strategy using composite system of metal organic framework (MOF) as bridge expanding SF's biomedical application is proposed. After developing the photocuring and bonding SF hydrogel, a MOF drug–loading system is utilized to enhance hydrogel's structural strength while endowing its antibacterial and angiogenic properties, yielding a multifunctional SF hydrogel. The synergy between the MOF and SF proteins at the secondary structure level gives this hydrogel reliable mechanical strength, making it suitable for conventional wound treatment, whether for closing incisions quickly or acting as adhesive dressings (five times the bonding strength of ordinary fibrin glue). Additionally, with the antibacterial and angiogenic functions getting from MOF system, this modified SF hydrogel can even treat ischemic trauma with cartilage exposure. This multiple modification should contribute to the improvement of advanced wound care, by promoting SF application in the production of tissue engineering materials.

## Graphical abstract

This study outlines endowing silk fibroin (SF) hydrogel with photocuring and adhesive properties. A metal organic framework (MOF) drug loading system leads the structural and functional modification. The MOF system accomplishes structural optimization by enhancing the secondary structure of SF and provides antimicrobial and angiogenic functions and structural–functional pluralistic modifying SF hydrogel for advanced wound care.

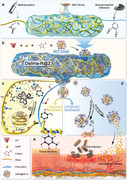

## 1 Introduction

As a natural biopolymer with a long history of and great applied energy, silk fibroin (SF) is actively used in the development of a variety of medical materials, especially in the area of skin health maintenance and injury care.^[^ ^1^ ^]^ SF wound dressings or wearables, prepared by processes like spinning are pre‐established,^[^ ^2^ ^]^ and additional auxiliary fixation is needed when dealing with skin injuries that have complex scenes (such as irregular‐shaped wounds), as they cannot adapt to deformation. Therefore, despite their flexibility, air permeability and affinity to the skin, they offer limited convenience and flexibility for application. Consequently, researchers are increasingly interested in developing SF hydrogels with good fluidity.^[^ ^3^ ^]^ Based on that, with the aid of a modification scheme for natural polymers such as gelatin, the characteristics of controllable light‐curing can be applied through the methacrylation of SF branch chains, to obtain plasticity in SF hydrogels.^[^ ^4^ ^]^ On the other hand, the biomimetic compound modification method inspired by mussels can endow hydrogels with reliable wet bonding abilities, as confirmed in our previous research.^[^ ^5^ ^]^ This enables the SF hydrogels to stabilize at the target skin site. Although the improved SF hydrogel‐state have advanced, rapid adaptability and biological adhesion, increased moisture reduces their structural strength^[^ ^6^ ^]^ and increases the possibility for bacterial growth,^[^ ^7^ ^]^ bringing about new threats to wound care.

Reliable mechanical strength is the premise for the practical application of light‐cured modified SF hydrogels in skin care, as well as the important guarantee for their biological‐bonding ability.^[^ ^8^ ^]^ Different from the conventional reinforcement from chemical crosslinking (such as glutaraldehyde or ethanol), it is a safe, bio‐friendly, and environmentally friendly scheme to toughen materials through the structure‐improving effect of nano‐fillers,^[^ ^9^ ^]^ and is suitable for the modification of hydrogels which need functional group protection.^[^ ^10^ ^]^ Being of great importance in the biomedical field, nano metal–organic frameworks (MOFs) play various roles as carriers, sensors or active additives,^[^ ^11^ ^]^ and are expected to become useful in the structural enhancement of SF hydrogels. Owing to the regular self‐assembly patterns between metals and ligands, different particles of the same MOFs can maintain a high degree of consistency in morphology and surface charge.^[^ ^12^ ^]^ Because of this property, when MOFs interact with natural polymers carrying various charges and branched groups, the microstructure of such hydrogels (e.g., chitosan hydrogels) would contain patterns that are more regular and their mechanical properties would be enhanced.^[^ ^8b^ ^]^ Similarly, for SF as a natural polymer, the potential‐stabilizing effect of MOFs on its microstructure may be quite beneficial, as it is reported that the more secondary protein structures are regularly arranged, the greater their mechanical strength.^[^ ^13^ ^]^ Therefore, using nano MOFs for the structural modification of SF hydrogels is a promising strategy for the creation of a good balance between feasible plasticity and flexible reliability.

Except during structure reinforcement, the challenges of bacterial infection and delayed healing are still faced during the application of such light‐cure adhesive SF hydrogels.^[^ ^14^ ^]^ Fortunately, as expected, MOF plays a role in dealing with such problems. The high specific surface area and porous structure of MOFs provide advantageous catalytic and drug‐loading platforms,^[^ ^15^ ^]^ which could broad the topological space of hydrogel to provide more functionalization possibilities. On the one hand, MOFs such as the zeolitic imidazolate framework‐8 (ZIF‐8) with photocatalytic effects can promote the controlled production of reactive oxygen species (ROS) in the presence of a light source, thereby endowing wearables with good antibacterial function.^[^ ^16^ ^]^ On the other hand, as nano‐carriers, MOFs can fulfill one or many functions such as promoting vascular healing, promoting mineralization and osteogenesis, or differentiation regulation through drug loading, to organize engineering materials functionally to promote biological activity.^[^ ^11^, ^17^ ^]^ Consequently, besides taking part in the structural reconstruction of SF as a nano‐filler, a MOF can be used as an effective functional modification element, to reduce the risk of bacterial infection induced by SF hydrogel enrichment. Moreover, it can result in functional modification by transporting active molecules to sites where they are needed, thereby promoting healing.

Herein, a modification strategy is proposed to revitalize SF‐based materials for skin wound care. Specifically, the light‐cure adhesive SF hydrogel (Dsilma) was obtained primarily through compound modification of the acryloyl group combined with 3,4‐dihydroxy‐D‐phenylalanine‐modified polyvinyl alcohol (PVA‐DOPA) as the bonding element. Then, ZIF‐8 was used to prepare a bridge system (R@Z) by loading 2‐deoxy‐D‐ribose (2dDR) as angiogenic molecules.^[^ ^18^ ^]^ For angiogenesis, 2dDR has reliable stability, low cost, and ease of incorporation into establishing wound care materials. Through the structural and functional modifications connecting R@Z to Dsilma, the multifunctional nursing gel (Dsilma‐R@Z) with a reliable structure, light‐controlled antibiosis functions and vascular healing‐enhancing capabilities was obtained, to address complex cases of skin damage. Benefiting from the interaction between MOF and the protein, the secondary structure of the proteins in Dsilma‐R@Z are arranged in a more orderly manner (the increased *β*‐folding). Consequently, this modification increases mechanical strength by 80% and bond strength by 40% (compared with the Dsilma without MOF modification). From the evaluation made during this study, in terms of functionality, Dsilma‐ R@Z can cater to conventional skin wounds with ease, whether as a medical glue to close the incision quickly, or as a flexible dressing to cover the defect perfectly. With the controlled production of ROS and drug release, Dsilma‐R@Z can protect a wound from the threat of bacteria with accelerating the tissue regeneration process. Furthermore, for dealing with complex trauma from ischemia and cartilage exposure, Dsilma‐R@Z can perform excellently, showing its co‐repair ability for soft and hard tissues and shortening the time for the healing process by about 1/3. The results above demonstrate that the structural and functional modification strategy from MOF bridging is a promising prospect for broadening the scope for the biological application of SF.

## 2 Results and Discussions

### 2.1 Synthesis and Characterization of Light‐Cured Adhesive SF Hydrogel

**Figure 1:**
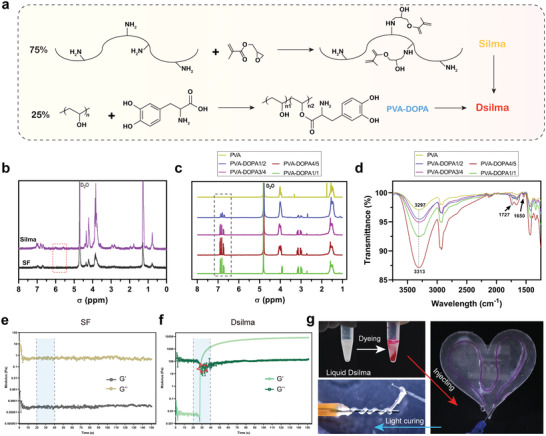
Synthesis and characterization of Dsilma. a) Illustrations displaying the method used to assemble Dsilma. b) ^1^H‐NMR spectra of SF and Silma. c) ^1^H‐NMR spectra of PVA‐DOPA polymers with different DOPA contents. d) FTIR spectra of different PVA‐DOPA polymers. e,f) The photocuring rheological properties of SF hydrogel and Dsilma hydrogel. g) The injectability, photocurability, and photoshaping properties of Dsilma.

In this study, methacrylated SF was engineered to have photocurable properties, making this material convenient for using in daily life or medical applications (Silma). Specifically, as shown in **Figure** **1**a, the amino groups on the side chains of water‐soluble SF were partially substituted by glycidyl methacrylate (GMA), thereby having the ability to be cross‐linked under light. Meanwhile, the addition of mussel‐inspired adhesive element, PVA‐DOPA enables the hydrogel closing the incision or covers the defect area quickly and stably without other wound dressings. This SF hydrogel with photocured and adhesion named Dsilma (D for DOPA and Silma for light‐cured water‐soluble silk protein). The formation of Silma and PVA–DOPA was confirmed using ^1^H‐nuclear magnetic resonance (^1^H‐NMR) spectra and Fourier‐transform infrared (FTIR). The vinylic peaks (*δ* 6.05 and 5.65 ppm) in the ^1^H‐nuclear magnetic resonance (^1^H‐NMR) spectra verified the conjugation of GMA to the SF chains (Figure 1b). Moreover, the peaks in the aromatic regions (*δ* 6.65, 6.72, and 6.78) demonstrated the successful introduction of DOPA in the PVA structure (Figure 1c) in which the highest degree of substitution comes to the PVA–DOPA 4/5 group, as previously reported (Table. S1, Supporting Information).^[^ ^5^ ^]^ Then, the existence of two vibration absorption peaks (1650 and 1727 cm^−1^) as well as the shift from 3297 cm to 3313 cm^−1^ are observed in Figure 1d; the appearance of those related covalent and hydrogen bonds demonstrated the successful synthesis of DOPA modification. According to this screening of DOPA group grafting efficiency shown above, PVA–DOPA4/5 was selected as the final adhesive modification ratio to obtain the Dsilma hydrogel. Further, in the rheological test, Dsilma presented a stable photocuring property, which could quickly obtain the storage modulus (G′) and then stabilization under the irradiation of blue light at 405 nm. In contrast, no photocrosslinking process was observed for SF hydrogels without methacrylate modification (Figure 1e,f). This light‐curd function of Dsilma benefited from the radical chain reaction of the grafted GMA groups under light irradiation.^[^ ^4^ ^]^ Moreover, as shown in Figure 1g, Dsilma also exhibited favorable injectability to fill serpentine thin tubes (Video S1, Supporting Information), and then could be taken out integrally after curing, showing good photoplasticity and photoshaping properties (Video S2, Supporting Information).

### 2.2 Synthesis and Characterization of MOF System

**Figure 2:**
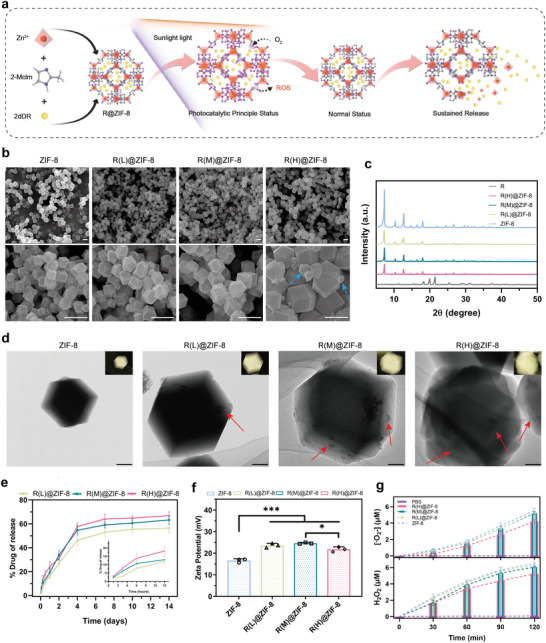
Characterization of the MOF system R@ZIF‐8. a) Schematic presentation of the fabrication of R@ZIF‐8 with the functions of generating ROS under sunlight catalysis and stable sustained releasing drug. b) SEM images. Scale bar: 1 µm. The blue arrows indicate particles of different sizes. c) XRD patterns of the ZIF‐8, R(L)@ZIF‐8, R(M)@ZIF‐8, and R(H)@ZIF‐8. d) TEM and elemental mapping images. The red arrows indicate the drug inside. Scale bar: 100 nm. e) Relative 2dDR released percent from R(L)@ZIF‐8, R(M)@ZIF‐8, and R(H)@ZIF‐8 samples. f) Zeta potential. *n* = 3 independent samples per group; *p = 0.017; ***p < 0.001. g) Variation in the amount of released ·O_2_‐ and H_2_O_2_ by different groups as a function of time.

To obtain the functions of nanofillers, antibacterial and healing promotion, the ZIF‐8 based MOF system loaded with different concentration of 2dDR were constructed and characterized using a previously modified “one‐step method.”^[^ ^8b^ ^]^ The drug loaded ZIF‐8 with low, middle and high concentration of 2dDR were marked as R(L)@ZIF‐8, R(M)@ZIF‐8 and R(H)@ZIF‐8. Considering the risk of excessive damage requiring to be controlled,^[^ ^19^ ^]^ light‐tunable ROS‐producing reactions were selected to specifically utilize the catalytic effect of nano‐ZIF‐8 to safely and controllably generate ROS under sunlight.^[^ ^16^ ^]^ This ensures that the wound is protected from bacterial damage, and it also has the characteristics of ease‐of‐use (by controlling the duration it is exposed to sunlight) for the general public. Besides, after comprehensive consideration of cost, storage and angiogenic efficacy, small‐molecule polysaccharide 2dDR was selected for activating revascularization.^[^ ^18^, ^20^ ^]^ All 2dDR loaded groups could generate ROS under sunlight catalysis^[^ ^16^ ^]^ as well as stable sustained release drug delivery,^[^ ^11a^ ^]^ as shown in **Figure** **2**a.

The morphology of the synthesized R@ZIF‐8 was observed using scanning electron microscopy (SEM), and classical dodecahedral particles, such as ZIF‐8 are observed for R@ZIF‐8 (Figure 2b); with increasing 2dDR content, R@ZIF‐8 gradually changes in particle size from ≈300 nm to ≈1 µm. X‐ray diffraction (XRD) patterns show the crystallinity of the ZIF‐8 phase in the R@ZIF‐8 with intense diffraction peaks at ≈7.3°, ≈10.4°, ≈12.7°, ≈14.6°, ≈16.4° and ≈18.0°, compared with the 2dDR phase with peaks at ≈20° and ≈21.5° (Figure 2c). The successful loading of 2dDR was confirmed using transmission electron microscopy (TEM) with related mapping, Bial's reaction, and drug release investigation. As shown in Figure 2d, the loaded 2dDR can be observed at the position indicated by the red arrow. It was verified that the 2dDR was located in the lattice rather than adsorbed on the surface, which were observed as additional particles in the TEM images without crystal‐type changes in XRD analysis (Figure 2c,d, and Figure S1a, Supporting Information). It can be observed using Bial's assay that the ZIF‐8 group shows the orange of Bial's reagent, and several groups of R@ZIF‐8 loading 2dDR show a distinct blue reaction which darkened with increasing drug loading content (Figure S1b, Supporting Information). Through the detection and calculation of these reaction reagents, we obtained the drug loading encapsulation efficiency (DLE) and drug loading capability (DLC), as listed in Table S1, Supporting Information. The drug loading encapsulation efficiency (DLE) and drug loading capability (DLC) of R@ZIF‐8 reaches 66.13 ± 2.35% and 12.93 ± 1.3%, respectively. Moreover, as shown in Figure 2e, the release features of 2dDR were consistent with the expectations. The release rates of several groups of R@ZIF‐8 were similar, and the drug release amount increased with increasing drug load. The increase in release rate did not match the increase in drug loading concentration likely because of the decrease in the specific surface area with increasing particle size.^[^ ^21^ ^]^

The change in particle size also affects the potential and catalytic reaction efficiency of R@ZIF‐8. As shown in Figure 2f, the zeta potentials of several groups of R@ZIF‐8 are significantly modified compared with those of ZIF‐8 (*p* < 0.05). Interestingly, the potential of R(H)@ZIF‐8 reduced than that of R(L)@ZIF‐8's and R(M)@ZIF‐8's own to its lower stability. When the 2dDR load approaching to the limit, the structure homogeneity of ZIF‐8 will be compromised, which was observed to be uneven in size and even some R(H)@ZIF‐8 lose its typical regular octahedron morphology (blue arrows in Figure 2b). As for photocatalytic efficiency, the ROS release represented by H_2_O_2_ and **·**O_2_‐ is shown in Figure 2g, and RL@ZIF‐8, RM@ZIF‐8 and ZIF‐8 exhibit similar ROS levels at 120 min (≈6.2 µm for H202, ≈5.2 µm for **·**O_2_‐), with that of RM@ZIF‐8 group being slightly lower at 30 min and then caught up. However, the ability of RH@ZIF‐8 to generate ROS was weaker, likely because the drug occupied ZIF‐8 and increased its particle size and reduced the specific surface area as well as available catalytic surface.^[^ ^22^ ^]^ Although the successful synthesis of all the planned R@ZIF‐8s was confirmed, loading with 2dDR and producing ROS under sunlight, it remained necessary to select a group with the most satisfactory overall performance. Because R@ZIF‐8 with different drug loadings all exhibited a slight pro‐proliferation effect (Figure S1c, Supporting Information), we selected R(M)@Z, which provided the highest drug loading (Figure 2e) under the premise of structural stability (Figure 2d,f) as well as a satisfactory ROS generation efficiency (Figure 2g). In addition, as shown in Figure S1d, Supporting Information, the interference of blue light (405 nm) on ROS generation was also excluded. The R(M)@ZIF‐8 was chosen into further hydrogel synthesis as “R@Z.”

### 2.3 Characterization and Strength Test of Dsilma‐R@Z

**Figure 3:**
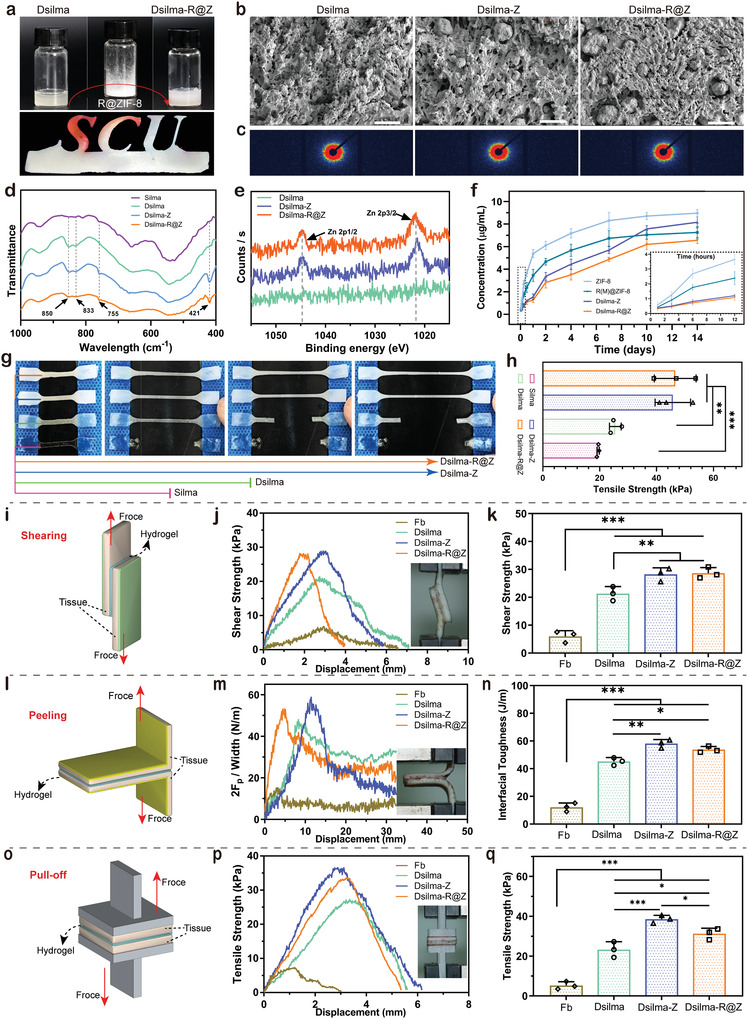
Characterization of the Dsilma‐based hydrogels. a) Composition of Dsilma‐R@Z and intricate patterns cast using it. b) SEM and elemental mapping images of Dsilma, Dsilma‐Z, and Dsilma‐R@Z. c) Exposure images of the hydrogel in the SAXS experiments. d) FTIR spectra of Dsilma‐based hydrogel and Silma. e) XPS spectra of zinc in Dsilma, Dsilma‐Z, and Dsilma‐R@Z. f) Cumulative concentration values of Zn ions released from ZIF‐8, R(M)@ZIF‐8, Dsilma‐Z, and Dsilma‐R@Z. g,h) Schematic images of tensile fracture and quantification of tensile strength testing. *n* = 3; i–k) Comparison of the shear strength of the Dsilma‐based hydrogels with the clinically applied fibrin glue via shear tests. *n* = 3. l–n) Comparison of the interfacial toughness of the Dsilma‐based hydrogels with the clinically applied fibrin glue via peel tests. *n* = 3. o–q) Comparison of the tensile strength of the Dsilma‐based hydrogels with the clinically applied fibrin glue via pull off tests. *n* = 3. For above statistics, *p < 0.05; **p < 0.01; ***p < 0.001.

For Dsilma‐R@Z, the core of its structural and functional Pluralistic modification is the loading of the above‐mentioned MOF system, R@Z. To achieve stable mechanical strength enhancement as well as optimization of biological functions, the participation of R@Z must be successful and uniform. R@ZIF‐8 was used to modify Dslima to prepare the Dslima‐R@Z hydrogel, which exhibited favorable photocurability and photoshaping properties (**Figure** **3**a), so could reproduce intricate patterns. For microscopic observations in dry form, a loose and porous morphology after lyophilization, similar to those of the Dslima and Dslima‐Z hydrogels (Figure 3b). Small‐angle X‐ray scattering results demonstrated that all three hydrogels exhibited anisotropic properties (Figure 3c) which declare the uniform loading of the MOF system.

As for the detection of nanoparticles, the Zn—N stretch mode at 421 cm^−1^ and the peak at 755 cm^−1^ were assigned to an out‐of‐plane bending of the ring, which confirms the presence of ZIF‐8 in Dsilma‐Z and Dsilma‐R@Z (Figure 3d).^[^ ^23^ ^]^ Elemental distribution mapping in Figure S2a, Supporting Information, EDS‐based element mass ratio in Figure S2b, Supporting Information, and X‐ray photoelectron spectroscopy (XPS) in Figure 3e both confirmed the presence of Zn in Dsilma‐Z and Dsilma‐R@Z, which have the potential to release Zn ions. The results of inductively coupled plasma‐optical emission spectrometry (ICP‐OES) further confirm this conclusion: pure ZIF‐8 and R@Z achieves the sustained release of zinc ions for ≈7 days, whereas Dsilma‐Z and Dsilma‐R@Z released zinc ions continuously after the 7th day and plateaued after the 10th day (Figure 3f). The drug release also exhibited a similar trend: unlike pure R@Z or simply adding the 2dDR into Dsilma, Dsilma‐R@Z still released 2dDR after the 7th day and gradually plateaued after 10 days, which could achieve multistage sustained release (Figure S2d, Supporting Information).

Swelling tests showed that the modification of MOF nanoparticles enabled Dsilma to perform more stable cured shape (Figure S2e, Supporting Information). Moreover, the following thermogravimetric analysis indicated that the successful addition of nanoparticles could enhance the thermal stability (Figure S2f, Supporting Information). When the temperature was lower than 300 °C, the mass loss of pure Dsilma hydrogel was ≈20% and those of Dsilma‐Z and Dsilma‐R@Z were less than 10%. The proportion of nanoparticles ZIF‐8 or R@Z in the hydrogel system was significantly less than 10%, indicating that their introduction significantly increased the thermal stability of Dsilma. In addition, as shown in Figure S2c, Supporting Information, the photocuring rheological properties are further explored. Under the irradiation of blue light (405 nm), both the two MOF modified Dsilma hydrogels were fully cured in ≈6 s (distance from the first dashed line to the star), indicating that the curing performance of Dsilma was not affected by increasing composition. The tensile strength tests showed that the cured pure Silma and the adherable Dsilma would break with a relatively small tensile force, while the cured Dsilma‐Z and Dsilma‐R@Z could bear a larger tensile force (Figure 3g). And the tensile strength of Dsilma‐Z and Dsilma‐R@Z increased by about 2 times compared with that without the MOF system added (Figure 3h). This may benefit from the enhancement effect of ZIF‐8 or R@Z as nanofillers.^[^ ^10^ ^]^ As wound caring materials, all Dsilma‐based hydrogels could stay in the wound area for enough time (Figure S2g, Supporting Information). In addition, Dsilma‐based hydrogels also show the reliability of their own compressive strength after curing (Figure S2h,i, Supporting Information). Therefore, with the incorporation of R@Z and ZIF‐8, the aforementioned properties enable the Dsilma system to have the wound adaptability for light management.

To comprehensively evaluate the adhesion strength of the hydrogels, simulating real‐world conditions, such as friction shedding, pulling off and tearing off, three types of forces were tested: 1) shear strength via shear tests (Figure 3i–k); 2) interfacial toughness via peel tests (Figure 3l–n); and 3) tensile strength via pull off tests (Figure 3o–q). Moreover, clinically applied fibrin hydrogel was used as a positive control. From the results, the three groups of biological hydrogels showed favorable adhesive strength under the three experimental models, reaching tens of kilopascals, greater than that of fibrin by a factor of ≈3–4. Dsilma‐Z and Dsilma‐R@Z performed better, and Dsilma‐Z performed slightly better in the peel and pull off tests. Adhesive properties’ test results confirmed that our hydrogel produced an effective biological soft tissue adhesion function under light control. Based on the above results, the integration of the Dslima‐R@Z biological hydrogel was successful. The modification of the MOF system enhanced Dsilma‐R@Z's ionic and drug multistage sustained release ability, multi‐stability, and mechanical properties.

### 2.4 Interaction Mechanism between Each Component of Dsilma‐R@Z

To explain the working principle of the rapid shape adaptability and bioadhesion ability of Dsilma‐R@Z, the synergistic and restraint relationship between proteins, polymers and nanoparticles in Dsilma‐R@Z was further explored. First, several rheological tests were performed to elucidate the hydrodynamic behavior of Dsilma‐R@Z in both injectable and cured crosslinked states. Strain‐dependent oscillatory rheology of the three hydrogels in crosslinked and injectable states both ideally exhibited broad linear viscoelastic regions except for moduli reduction at significantly high strains (>10% shear strain), illustrating an excellent processing regime and shear‐thinning behavior (Figure S3a, Supporting Information). For steady shear measurements, the non‐Newtonian behavior of shear‐thinning was further demonstrated, exhibiting a gradual decrease in viscosity across a wide range of shear rates (Figure S3b, Supporting Information). The frequency sweep profiles showed that both the storage (G′) and loss moduli (G⁗) were observed to gradually increase with increasing oscillation frequency (Figure S3c, Supporting Information). It is also observed that Dsilma‐R@Z and Dsilma‐Z exhibit higher resistances to modulus change compared with Dsilma, indicating they exhibit better stabilities when subjected to external forces. Notably, Dsilma‐Z exhibited a higher initial modulus than Dsilma‐R@Z, which was also observed in a few previous mechanical tests. From dynamic time sweep, the crosslinked biohydrogels exhibited a distinct gel state (G′ > G⁗), injectable biohydrogels showed a flowing sol state (G⁗ > G″), and neither changed with time (Figure S3d, Supporting Information). It was also observed that ZIF‐8 and R@Z significantly reduced the loss factors (tan□ = G⁗/G″) of Dsilma in the injectable state, indicating that the elastic properties of Dsilma were enhanced. Additionally, all Dsilma‐based hydrogels presented self‐healing behavior at injectable state, following with the MOF enhanced groups had better damage resistance than Dsilma groups after light curing (Figure S3e, Supporting Information). Owing to the moderate viscosity and fluidity, Dsilma‐R@Z in the liquid state could gradually fill subtle skin defects while ensuring its relatively stable retention in the wound area, which is the basis for its adaptation to the wound.

**Figure 4:**
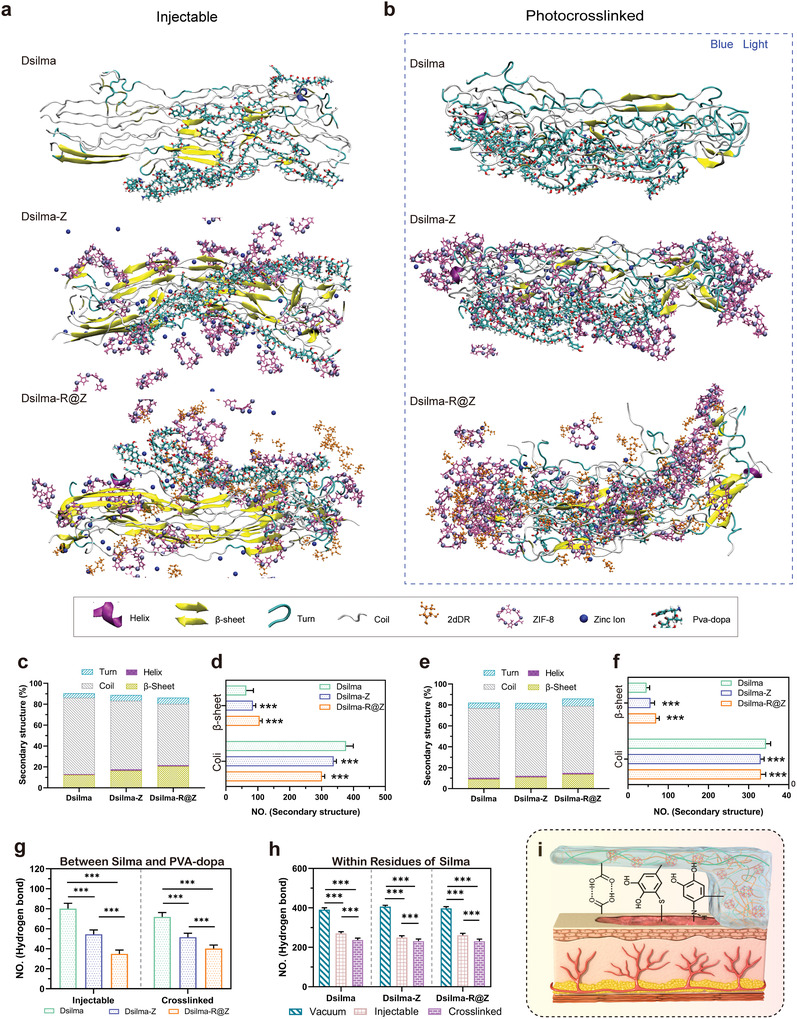
Molecular simulation of the Dsilma‐based hydrogels in different states. a) Representative snapshots of the Dsilma‐based hydrogels in injectable state. b) Representative snapshots of the hydrogels in photocrosslinked state. c) Secondary structure proportion of Silma in different groups in injectable state. d) Number of *β*‐sheets and coils of the hydrogels in the injectable state. *n* = 401; ***p < 0.001. e) Secondary structure proportion of Silma in different groups under photocrosslinked state. f) Number of *β*‐sheets and coils of the hydrogels in photocrosslinked state. *n* = 401; ***p < 0.001 versus Dsilma. All data are Mean ± S.D. g) Number of Hbonds formed between the Silma and PVA‐DOPA in different hydrogels in injectable state and photocrosslinked state. *n* = 401; ***p < 0.001. h) Number of Hbonds formed within residues of Silma in different hydrogels in vacuum, injectable, and photocrosslinked state. *n* = 401; ***p < 0.001. i) Schematic overview of the interactions between the Dsilma and soft tissue.

Moreover, to elucidate the mechanism of the significant changes in the adhesive strength, compressive strength, and rheological behavior of pure Dsilma with the introduction of ZIF‐8 or R@Z, all‐atom molecular dynamics (AAMD) simulations were performed. AAMD simulations were performed on Dsima‐based groups of three states: i) injectable state; ii) photocrosslinking state; and iii) vacuum state (storage state) (**Figures** **4**a,b, and Figure S3f, Supporting Information). The simulations showed that ZIF‐8 and R@Z affected the structural relationships within the protein by increasing the ratio of the strong secondary structure (*β*‐sheets) and decreasing the amount of fragile coil structures.^[^ ^13^ ^]^ In particular, in the injectable state (Figure 4a), the proportion/number of *β*‐sheets in each group were ≈12.4%/64, ≈16.4%/85 and ≈20%/106, respectively, and the corresponding ratios of the coils were ≈73%/377, ≈65.6%/338 and ≈58.4%/301 (Figures 4c and 4d). Then, a similar trend was also observed in the photocrosslinked state, where Dsilma‐Z and Dsilma‐R@Z increased the *β*‐sheets with fewer coils in the secondary structure calculation (Figure 4e,f). Moreover, the relationship between proteins and polymers also significantly changed with the addition of ZIF‐8 or R@Z. Whether in the injectable or photocrosslinked state, the number of hydrogen bonds between Silma and PVA–DOPA significantly reduced with the addition of ZIF‐8 and R@Z (Figure 4g). Additionally, the number of hydrogen bonds was also significantly affected by the varying state, which clearly indicates the trait changes in hydrogel. The number of hydrogen bonds in all the groups within the Silma residues were the highest in the vacuum state, and subsequently reduced when water molecules were incorporated in the injectable state, and appeared significantly lower after the occurrence of curing cross‐linking (photocrosslinked state) (Figure 4h). Furthermore, according to previous study,^[^ ^8^, ^24^ ^]^ the adhesive mode between Dsilma and soft tissue predominantly relies on hydrogen and covalent bonding between the catechol groups on PVA–DOPA and soft tissue (Figure 4i).

Based on the AAMD simulation results, the introduction of ZIF‐8 and R@Z changed the secondary structure composition of Silma and the number of hydrogen bonds between PVA–DOPA and Silma. Moreover, a certain relationship was observed between the two: owing to the protective effect of ZIF‐8 and R@Z on the reduction in hydrogen bonding between PVA–DOPA and Silma, the regular secondary structure formed in Silma was promoted, which substantially increased *β*‐sheets and distinctly decreased random coils.^[^ ^13^, ^17^ ^]^ The cooperation of various components at the protein secondary structure level finally facilitated higher mechanical and adhesive strengths of Dsilma‐R@Z, thereby enhancing wound protection compared with pure Dsilma hydrogel.

### 2.5 In Vitro Antibacterial Ability Evaluation

**Figure 5:**
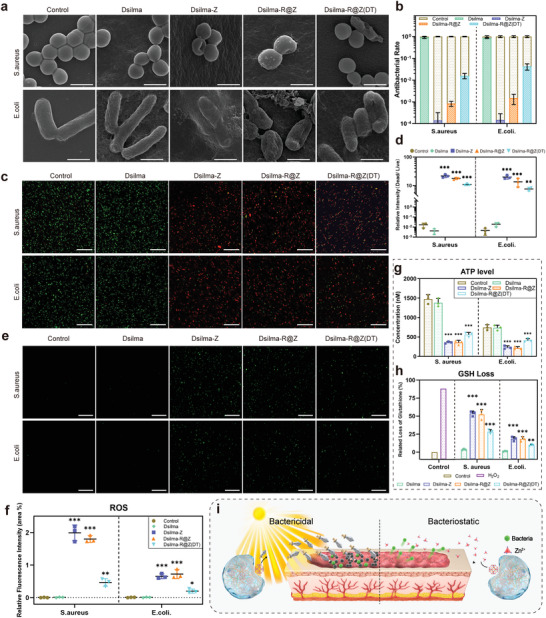
In vitro antibacterial ability. a) SEM images of *S. aureus* and *E. coli* treated with normal saline, Dsilma, Dsilma‐Z, Dsilma‐R@Z, and Dsilma‐R@Z (DT). Scale bar: 1 µm. b) Comparison of antibacterial rates of different treatments against *S. aureus* and *E. coli*. c) Confocal fluorescence images of *S. aureus* and *E. coli* stained with Calcein‐AM/PI dyes after different treatments. Scale bar: 30 µm. d) The relative fluorescent intensity (dead/live) in *S. aureus* and *E. coli* with different treatments. Scale bar: 30 µm. *n* = 3; **p = 0.0021, ***p < 0.001. e) Confocal images of ROS level in *S. aureus* and *E. coli* cells after incubation with normal saline, Dsilma, Dsilma‐Z, Dsilma‐R@Z, and Dsilma‐R@Z (DT). f) Relative ROS levels in *S. aureus* and *E. coli* cells with different treatments. *n* = 3; *p = 0.0357, **p = 0.0046, ***p < 0.001 versus Control. g,h) Quantitative statistics of ATP level and GSH loss of *S. aureus* and *E. coli* cells after different treatments. *n* = 3; **p = 0.0054, ***p < 0.001 versus Control. i) Schematic diagram for the antibacterial ability of Dsilma‐R@Z, which has both bactericidal effect of sunlight control as well as long‐term bacteriostatic effect of no light automatic control.

Infection control is key for wound protection and is a prerequisite for wound healing. The injured area should be protected from bacterial infection for a stable microenvironment, and the damage caused by the inflammation of the body may be reduced, after which the tissue repair process can be initiated.^[^ ^14^ ^]^ Herein, we evaluated the in vitro antibacterial properties of a series of Dsilma‐based hydrogels. As shown in **Figure** **5**a, both *Staphylococcus aureus* (*S. aureus*) and *Escherichia coli* (*E. coli*) cultured on Dsilma‐Z and Dsilma‐R@Z exhibit distinct depressions, shrinkage deformations as well as fractured morphologies, and the bacteria cultured with the 3‐day delay immersion solution of Dsilma‐R@Z (DT) also shrunk slightly. Then, the antibacterial test showed that (Figure 5b and Figure S4a, Supporting Information) the antibacterial rates of Dsilma‐Z and Dsilma‐R@Z were both greater than 99.9%, whereas that of Dsilma‐R@Z (DT) was slightly lower than that rate. The same antibacterial advantage was also observed in the zone of inhibition test (Figure S4b,c, Supporting Information). The above trends suggested that the MOF system in Dsilma‐R@Z seems to still exert a certain antibacterial effect after the short‐term photocatalytic‐driven (≤2 h) antibacterial effect was completed. The following live/dead staining assay and its fluorescence quantification statistic further confirmed this trend, per Figure 5c,d, thereby indicating the advantages of Dsilma‐Z and Dsilma‐R@Z to eliminate bacteria (the count of dead bacteria was much more than that of live bacteria) compared with the control and Dsilma groups. Additionally, the bactericidal effect of Dsilma‐R@Z (DT) was slightly inferior, and more live bacteria was observed; however, dead bacteria remained the majority. The live/dead test results are consistent with the SEM and colony‐count results, indicating that the Dsilma hydrogel containing ZIF‐8 or R@Z exhibits a strong bactericidal effect under controllable photocatalysis, whereas the solution of Dsilma‐R@ Z without light treatment also facilitated antibacterial activity. Considering the degradation and low concentration of ROS generated by short‐term photocatalysis, ROS may be greatly attenuated after the immediate process. Therefore, when using the eluate of Dsilma‐R@Z left standing for 3 days to fight bacteria, the role of other possible antibacterial components should be considered, such as the Zn^2+^ slowly released from R@Z.^[^ ^8b^ ^]^

To further elucidate the antibacterial mechanisms as well as explore the possible long‐term antibacterial mechanism, we used the DCFH‐DA probe to monitor the ROS level (Figure 5e,f, and Figure S4d,e, Supporting Information). It was observed that the high levels of ROS (green fluorescence spots) were produced in *S. aureus* and *E. coli* under the treatment of Dsilma‐Z and Dsilma‐R@Z; contrastingly, the ROS level produced by the Dsilma‐R@Z (DT) and Dsilma‐Z(DT) groups were lower, but remained higher than those of the control, Dsilma or Dsilma(DT) groups. ATP is an important energy molecule that characterizes the normal respiratory chain activity of bacteria, and its reduction is an important indicator of the ROS destroying bacteria.^[^ ^25^ ^]^ As shown in Figure 5g and Figure S4g, Supporting Information, the trend of ATP levels is negatively correlated with the ROS levels in each group: compared with the control and Dsilma groups, for two types of bacteria treated with Dsilma‐Z and Dsilma‐R@Z, the ATP levels significantly decreases. Besides, for delay time groups (Dsilma‐R@Z [DT] and Dsilma‐Z [DT]), the ATP also decreased to a smaller extent. Glutathione (GSH) could be oxidized into GSSG by present radicals and is an important reserve for defense against ROS, so the degree of its loss indicates the ROS damage level.^[^ ^26^ ^]^We detected that the two kinds of bacteria treated with Dsilma‐Z and Dsilma‐R@Z exhibited substantial GSH loss, the Dsilma‐R@Z (DT) and Dsilma‐Z (DT) group also exhibited some depletion, whereas the control and Dsilma groups exhibited virtually no GSH loss (Figure 5h and Figure S4f, Supporting Information), so this trend was consistent with the ROS results. According to the above results, it can be inferred that short‐term controllable sunlight can catalyze Dsilma‐R@Z to generate ROS to eliminate a large number of bacteria that adhered at the initial stage and complete wound cleaning to protect the wound surface. This model of on‐demand antimicrobial therapy is beneficial for wound healing.^[^ ^27^ ^]^ For infected wounds, ROS generated using sunlight‐activated R@Z or ZIF‐8 in Dsilma can accumulate in bacteria (Figure 5e,f), effectively interfering with bacterial mitochondrial activities and depleting their reduction protective protein (Figure 5h) to eliminate bacteria.^[^ ^25^ ^]^ Additionally, this hydrogel also facilitates a delayed protective effect unrelated to short‐term ROS, which may inhibit the growth of bacteria through the slow release of Zn^2+^ and continuously protect the wound, which is likely consistent with our previous report that Zn^2+^ has an antibacterial effect^[^ ^8b^ ^]^ (Figure 5i).

### 2.6 Biocompatibility Evaluation and In Vitro Angiogenesis Assay

Preclinical safety assessment is among the most important prerequisites for wound‐treatment materials before clinical translation.^[^ ^28^ ^]^ Therefore, a series of biosafety studies were performed, including in vitro cytotoxicity, survival analysis, in vivo degradation and in vivo histopathology of skin tissues and major organs, were performed. For early cell adhesion, the Dsilma‐based hydrogel groups exhibited acceptable safety profiles, on which fibroblasts and vascular endothelial cells were able to successfully colonize after ≈2 h (Figure S5a, Supporting Information). Further, the fluorescence images in Figure S5b, Supporting Information, shows that the cells could also perform typical proliferation‐related physiological activities, including the production of cell protrusions and the extension of pseudopodia, which demonstrates the biosafety of the hydrogel prepared in this study. The cell growth was more quantitatively shown in the CCK‐8 assay (Figure S5c, Supporting Information). In particular, at day 5, L929s and HUVECs cocultured with hydrogels containing R@Z and ZIF‐8 components exhibited different degrees of proliferation activation, and this trend was not distinct at Day 1 or 3. Moreover, considering the possible oxidative damage caused by high energy light,^[^ ^29^ ^]^ we conducted a relevant ROS test under by strictly controlling the blue light irradiation time. As shown in the flow cytometry fluorescence quantitative results in Figure S5d, Supporting Information, 15 s of blue light irradiation did not cause any observable damage to the experimental groups compared with the negative control, which is consistent with the reported light absorption band of ZIF‐8,^[^ ^16^ ^]^ confirming the biosafety of Dsilma‐R@Z.

For the activation of angiogenesis in vitro, we adopted a typical scratch assay and Transwell assay for characterization.^[^ ^30^ ^]^ As shown in Figure S5e, Supporting Information, after 24 h of recovery, the width of the “scar” narrowed most significantly in the group affected by the Dsilma‐R@Z precipitate. Further quantitative statistics showed that the HUVECs treated with the complete Dsilma‐R@Z group exhibited the most rapid healing speed compared with other hydrogel groups without or only integrating the pure nano‐ZIF‐8 (Figure S5f, Supporting Information). In addition, the quantitative characterization results of the Transwell‐based chemotaxis assay also support the distinct advantage of R@Z, and the addition of nano‐ZIF‐8 also appears to have a slightly positive effect on the chemotaxis of vascular endothelial cells (Figure S5g, Supporting Information). Therefore, all the biocompatibility researches demonstrated a high biosafety of Dsilma‐R@Z for both fibrous and blood vessels tissue. Addtionally, the advanced activation for angiogenesis of Dsilma‐R@Z (Figure S5h, Supporting Information) indicated its significant potential for achieving wound curing by accelerating wound healing.

In addition to in vitro cytocompatibility studies, in vivo biosafety was further evaluated in Sprague Dawley rats. We injected and cured the hydrogel groups into the subcutaneous space on the back of rats them to explore the biosafety as well as degradability of the hydrogels in vivo (Figure S6a, Supporting Information). As shown in Figure S6b, Supporting Information, compared with the TritonX‐100‐containing positive control group, Dsilma‐R@Z and other Dsilma‐based hydrogels could be considered as non‐hemolytic and safe in direct contact with blood. Further results for the skin tissue at the surgical implant site indicate that there was no significant inflammation, necrosis or metaplasia in contact with the hydrogels for 21 days compared with the normal tissue, and the Dsilma‐based hydrogel was completely degraded (Figure S6c, Supporting Information). Further evaluation was performed to detect the histocompatibility of the hydrogel with major organs (heart, liver, spleen, lung, and kidney), and the H&E staining results (Figure S6d, Supporting Information) showed no distinct tissue damage or pathological change in any of the major organs after implantation for 21 days.

### 2.7 Hemostasis, Adhesion, and Treatment for Conventional Incisions

For providing effective protection for skin wounds, a portable wound caring material that can be quickly used to stop bleeding, resist infection, and bond to wound stably is an attractive prospect.^[^ ^31^ ^]^ Therefore, we explored the ability of Dsilma‐R@Z to protect wound in emergency situations through its rapid adhesion and antibacterial properties. The inevitable bleeding after being injured makes hemostasis the primary aim of wound management;^[^ ^32^ ^]^ therefore, various Dsilma‐based hydrogel groups were used to treat rapid hemorrhage caused by a tail docked in Sprague Dawley rats (Figure S7a, Supporting Information). Then, we injected a hydrogel at the truncation and cured for evaluation of hemostatic function for active bleeding in the rat tail. After 15 min of observation, Dsilma, Dsilma‐Z and Dsilma‐R@Z hydrogels significantly prevented the blood exudation from the fracture of the rat tail, whereas the rats in untreated group lost blood weight over 20 times that of these gel‐treated groups (Figure S7b and Video S3, Supporting Information). These results demonstrate the excellent hindering of wound blood loss using this class of Dsilma multifunctional therapeutic materials.

Then, severe incisions using *S. aureus*‐infected Sprague Dawley rats (on the back) were treated with Dsilma, Dsilma‐Z and Dsilma‐R@Z hydrogels, or no treatment, and each group received an equivalent solar lighting simulation for 2 h (Figure S7a, Supporting Information). As shown in Video S4, Supporting Information, under light, Dsilma‐R@Z could adapt to the shape of the incision and then quickly and stably close it; it was also able to withstand skin pulling and squeezing. As shown in Figure S7c, Supporting Information, from Day 3 after surgery, compared with those in the untreated control group, the wounds of the rats in each bioadhesive hydrogel treated group remained substantially closed, and the damage areas were significantly smaller (Figure S7g, Supporting Information). This showed those Dsilma have played a good role in bonding tissue. However, the histological analysis using H&E showed that the therapeutic effect of the hydrogel containing the R@Z nano‐drug loading platform was significantly superior to those of the other groups (Figure S7d, Supporting Information). In particular, compared with the fragmented soft tissue and large numbers of neutrophils shown in the untreated or Dsilma‐only containing samples, the subcutaneous tissue of the incision treated using Dsilma‐R@Z exhibited a continuous structure and less inflammation, and a thin keratinized layer growing along the cut, which was not observed in other groups. Further histological results on Gram‐positive bacteria shown in Figure S7e, Supporting Information, verified the photocatalytic antibacterial advantages of R@Z and nano‐ZIF‐8, wherein the bacterial infection in the incisions treated using nanoparticle‐loaded hydrogels were effectively curbed, and numerous bacteria (yellow arrows) remained in the Dsilma and control group. Moreover, immunofluorescence staining for vascular endothelial growth factor A (VEGFA, red) and tumor necrosis factor‐alpha (TNF‐*α*, green) demonstrated that Dsilma‐R@Z hydrogel resisted bacterial infection to reduce the expression of inflammatory factors^[^ ^33^ ^]^ through its photocatalytic properties as well as accelerated early revascularization by significantly mobilizing vascular growth factors,^[^ ^34^ ^]^ further demonstrating its ability to protect and promote wound healing (Figure S7f–j, Supporting Information).

### 2.8 In Vivo Therapeutic Efficacy for Treating Atypical‐Circular Skin Defects

**Figure 6:**
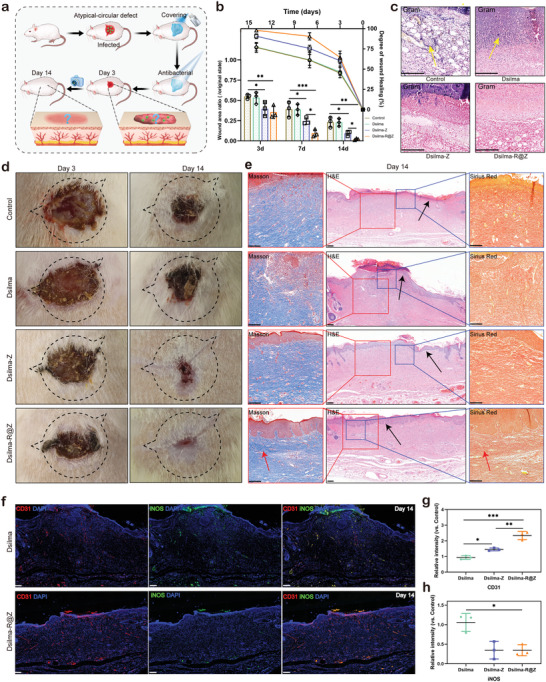
Treatment of atypical‐circular skin defects. a) Schematic representation of the skin defects model. b) The ratio of wound area to original state and wound healing curve at 3, 7, and 14 days. *p < 0.05;**p < 0.01; ***p < 0.001. *n* = 3. c) Gram staining of the wound area at Day 3, yellow arrows indicate the bacteria causing the infection. d) Digital images of the wound after 3 and 14 days with no hydrogel treatment (control), and under treatment of Dsilma, Dsilma‐Z as well as Dsilma‐R@Z. The initial wounds' area was marked by dotted areas. e) Masson staining, H&E staining, and Sirius Red staining of the wounded skin at Day 14. The black arrows indicate keratinized layers while the red arrows indicate regular collagen. f) Immunofluorescence staining of platelet endothelial cell adhesion molecule‐1 (CD31, red), inducible nitric oxide synthase (iNOS, green), and nuclei (DAPI, blue) for the wounds at Day 14. g,h) Relative CD31 and iNOS fluorescence intensity quantification versus Control group. *p <0.05;**p = 0.0019; ***p < 0.001. *n* = 3. All above scale bars equal to 200 µm.

Wounds with large skin defects often face a higher risk of infection and longer healing durations and thus, they need more complete coverage and healing promotion.^[^ ^32c^ ^]^ To provide more convenient wound protection, a few researchers are working to develop biomaterials that can quickly close wounds. For instance, the uncontrolled‐curing biological hydrogels modified using aldehydes, transglutaminases and cyanoacrylate groups as well as a few medical patches based on polymer adhesives, such as poly(l‐lactide‐co‐ɛ‐caprolactone), have been used to close wounds.^[^ ^5^, ^8^, ^35^ ^]^ However, these solutions have limitations, such as uncertain toxicity, limited or slow reaction times, inability to cover large defect areas, or the requirement of multiple trimmings to match the shape of the wound. In order to prove the function of rapid wound adaptation and treatment, we tried to investigate the therapeutic efficacy of Dsilma‐R@Z hydrogel for adhesively covering and vascularized healing. Several atypical‐circular defects were prepared on the back of Sprague Dawley rats (Figure S8d, Supporting Information). They were also exposed into an infectious microenvironment, and subsequently sprayed with hydrogels (Dsilma, Dsilma‐Z, and Dsilma‐R@Z) under light for coverage and treatment. We observed the wound sizes for 14 days, and the wound images and specimens at the early (Day 3) and late (Day 14) postoperative stages were recorded to assess the extent of wound infection and recovery, respectively (**Figure** **6**a). Owing to its excellent flow and adhesion, Dsilma‐R@Z and other Dsilma hydrogels were ejected as a liquid column to adapt and cover the wound with acceptable strength and stability after light curing (Video S5, Supporting Information). Simultaneously, the strong adhesive effect of the Dsilma‐based hydrogel enabled it to be riveted stably with the wound and surrounding skin tissue (Video S6, Supporting Information).

In the initial stage of wound recovery, as shown in Figure 6b,d, Dsilma‐R@Z and Dsilma‐Z significantly facilitated wound healing, resulting in a smaller residual wound area. This healing advantage may arise from the effective resistance to bacteria, showing fewer bacteria in the Gram stain image in Figure 6c, compared with the Dsilma‐only and control groups. The Dsilma‐R@Z and Dsilma‐Z groups showed less subcutaneous hemorrhage and neutrophil infiltration in the corresponding H&E and Masson histological analysis, which also indicated that they effectively reduced infectious inflammation. The above trend confirmed that this MOF system, R@Z, could indeed improve the antibacterial ability of SF hydrogels. Additionally, the composite fluorescent staining results of VEGFA and tumor necrosis factor‐alpha (TNF‐*α*) also confirmed the above trend, wherein Dsilma‐R@Z exhibited certain vascular activation and anti‐inflammatory effects (Figure S8a–c,e,f, Supporting Information) in the early stage. At the same time, the results of tissue recovery suggested that short‐term controlled use of ROS does not have an unacceptably negative impact on wound healing. Owing to the acute response to trauma, wound tissue repair does not immediately begin but first processes the exposed partially necrotic cells,^[^ ^19^, ^36^ ^]^ thereby providing a safe window for the short‐term (within 2 h) bactericidal effect of the ROS period without damaging the regenerated tissue.

**Figure 7:**
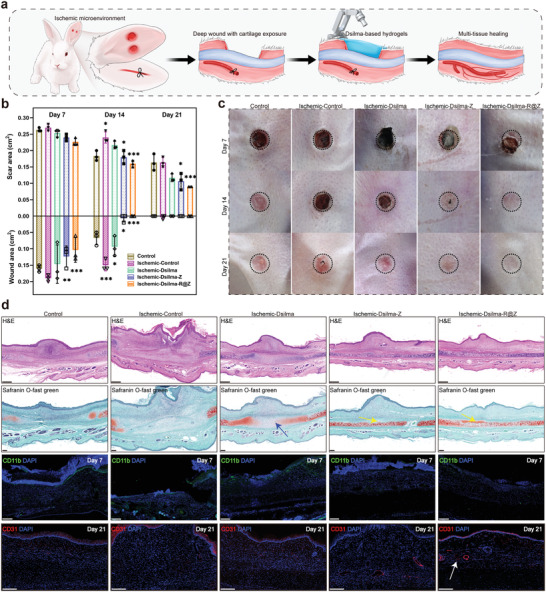
Treatment of ischemic traumas with hard tissue exposure. a) Schematic representation of the ischemic trauma model. b) The wound or scar area at 7, 14, and 21 days. *p < 0.05; **p < 0.01; ***p < 0.001. *n* = 3. c) Digital images of the wound after 7, 14 and 21 days with no hydrogel treatment (control), and under ​treatment of Dsilma, Dsilma‐Z as well as Dsilma‐R@Z. The initial wounds' area was marked by dotted circle. d) H&E (scale bar = 500 µm), Safranin O‐fast green, immunofluorescence staining of differentiation antigenic cluster molecule 11b (CD11b, green) and nuclei (DAPI, blue) for wounds at Day 7, immunofluorescence staining of platelet endothelial cell adhesion molecule‐1 (CD31, red) and nuclei (DAPI, blue) for the wounds at Day 21, scale bar = 200 µm. The blue arrow indicates immature cartilage while the yellow arrows indicate mature cartilage. The white arrow indicates micro vessels.

Regarding the wound recovery in the later stage, according to the statistics and photos for 14 days shown in **Figure** **7**b,d, it can be observed that additional to the wounds treated with Dsilma‐R@Z exhibiting the fastest healing rate, Dsilma‐Z also exhibited treatment effects. In particular, the best‐performing R@Z hydrogel group successfully closed the wound by 97.950 ± 0.985%, whereas 90.671 ± 2.915%, 77.174 ± 4.715% and 76.904 ± 6.168% of Dsilma‐Z, Dsilma and untreated group, respectively. Moreover, in the following histological analysis, the most mature collagen remodeling (red arrows in Figure 6e), and keratinized layer with epithelial spikes were observed in the soft tissue in Dsilma‐R@Z group compared with the other three groups (black arrows in Figure 6e), whose keratinized layer appeared weak or smooth. In addition, fluorescent histological staining based on CD31 and iNOS was used to evaluate the deeper manifestations of the wound–repair effect.^[^ ^11b^ ^]^ As shown in Figure 6f and Figure S8g, Supporting Information, the R@Z loads directly determine the therapeutic efficacy of Dsilma‐based hydrogel on skin defects, through which Dsilma is endowed with an antibacterial ability to anti‐inflammatory (down‐regulated iNOS) as well as pro‐angiogenic capacity (up‐regulated CD31). Moreover, although it did not show the ability to up‐regulate Vegfa in the early stage, the fluorescence quantification relative to the untreated group also demonstrated the superiority of Dsilma‐R@Z in angiogenesis promotion of the wound (Figure 6g,h), suggesting that ZIF‐8 may have a potential biological promotion mechanism.

### 2.9 In Vivo Therapeutic Efficacy for Treating Ischemic Complex Trauma

**Figure 8:**
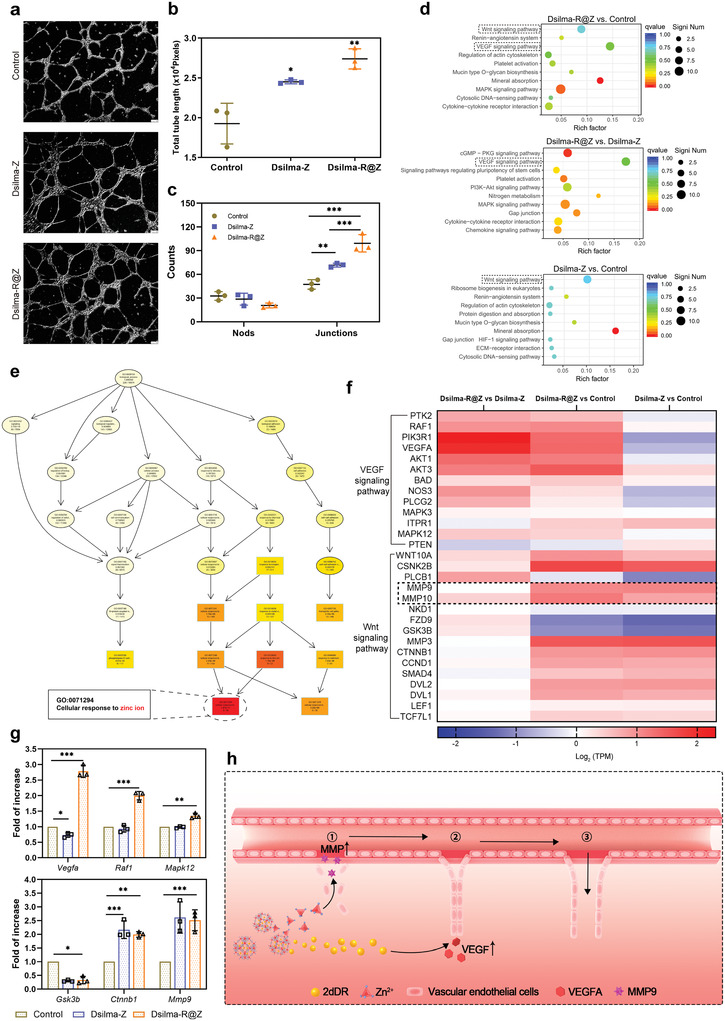
Synergistic angiogenesis mechanism of Dsilma‐R@Z on gene expression level. a) Images of tube formation simulation experiments under Dsilma‐Z and Dsilma‐R@Z treatments (scale bar = 100 µm). b,c) Statistics of tube lengths, junctions, and nods. *n* = 3. *p = 0.0188; **p < 0.01; ***p < 0.001. d) Representative top 10 upregulated KEGG signaling pathways. e) GO analysis of Dsilma‐Z compared to controls to explore the role of ZIF‐8. f) Heat map showing the fold change of selected gene expression. g) Validation of expression levels of some genes in VEGF and Wnt signaling pathways by RT‐PCR. *p < 0.05;**p < 0.01; ***p < 0.001. *n* = 3. h) Synergistic angiogenesis mechanism of Dsilma‐R@Z.

Unlike ordinary skin wounds that can immediately be treated, damages in the weak parts of the subcutaneous tissue, such as limbs, joints, nose and ears, often face high tension, ischemia, deep tissue exposure and increased risk of hard tissue resorption, which significantly increases the difficulty of wound repair.^[^ ^37^ ^]^ Therefore, we built a high‐tension wound model (full thickness of skin removed without damaging the cartilage structure) with bone exposure under ischemic microenvironment (**Figure** **8**a, and Figure S9a,b, Supporting Information), using which we evaluated the therapeutic efficacy of Dslima‐R@Z with the assistance of a self‐developed simple surgical robot (Figure S9c, Supporting Information). Figures 8b and 8c show the trauma recovery during the 21‐day postoperative monitoring, where the “wound area” refers to the area not covered by the keratinized skin, and the immature keratinization area is represented as the “scar area.”

It could be observed that the microenvironment ischemia (ischemic‐control group versus control group) significantly hindered the shrinkage of wounds and scars, particularly during early wound healing at Day 7, when the collateral revascularization was not completed yet. Conversely, owing to revascularization‐activating components, Dsilma‐R@Z could decrease the healing time of the ischemic wound area by ≈7 days under the same ischemic microenvironment, facilitating a faster process for scar elimination.^[^ ^37^, ^38^ ^]^ Moreover, the barrier protection provided by the hydrogel and the potential biological activity of ZIF‐8 was considered, and the Dsilma and Dsilma‐Z hydrogels also showed a certain therapeutic efficacy (Figure 8b,c).

Then, the repair details of multiple tissues including soft tissue, vascular, and cartilage repair were histologically analyzed (Figure 8d and Figure S9d, Supporting Information). As shown in the H&E and Safranin O fast green staining images, the cartilage tissue protected by Dsilma‐R@Z and Dsilma‐Z hydrogels was not exposed to resorption, showing intact cartilage boundaries as well as repaired tissue (yellow arrows). Meanwhile, although the newly repaired cartilage was significantly immature (blue arrow in Safranin O fast green staining), the protective effect of Dsilma for the cartilage remained significantly superior to that of the control or ischemic control groups, where the cartilage under the wound was absorbed. In addition, as shown in Figure S9d in the Supporting Information, subcutaneous wounds control or ischemic control groups also exhibited more disorganized repairing collagen (red arrows in Masson staining) because of exposed directly to external damages, which is consistent with the trend of scar residue. As for the control of early inflammation, the inflammatory responses were lower under Dsilma‐R@Z or Dsilma‐Z treating, which benefit from the dual advantages of Zn^2+^ as antibacterial and anti‐inflammatory (Figure 8d, and Figure S7d and S9e, Supporting Information). For the therapeutic efficacy of wound healing, the revascularization level was characterized using VEGF and CD31 immunofluorescence staining. As shown in Figure 8d and Figure S9d, Supporting Information, early angiogenic factor activation and clustered micro vessels (white arrow) are observed under the treatment of Dsilma‐R@Z, thereby leading to enhanced revascularization, with Dsima‐Z also exhibiting a certain positive effect on angiogenesis (Figure S9f, Supporting Information). The reason of Dsilma‐R@Z protecting exposed cartilage from resorption may be multi‐faceted: 1) reducing cartilage resorption by direct barrier action; 2) blocking early infection and inflammation; and 3) promoting regional revascularization to accelerate the regeneration of already stimulated cartilage.^[^ ^39^ ^]^

Vascularized tissue healing is a tissue regeneration model guided by revascularization, which is an important physiological process in the body to repair damage.^[^ ^37^ ^]^ Whether the tissue is soft or hard, numerous studies have reported the synergistic crucial role of vascularized repair, and various tissue engineering materials have been prepared for this design. For example, Mg‐based materials, nanoparticles or bioglass, were often used to activate angiogenesis around the bone tissue, facilitating a beneficial microenvironment for bone reconstruction as well as other tissues’ healing.^[^ ^11^, ^40^ ^]^ Inspired by this, for silk fibroin‐based hydrogels multi‐modified by R@Z or ZIF‐8, perhaps the designed 2dDR was not the only role in promoting angiogenesis to repair soft and hard tissues; the Zn^2+^ also was an important assistant. Those the above results on wound repair involving multiple tissues in complex situations reiterated the therapeutic efficacy of using the Dsilma‐R@Z hydrogel, which also showed some advantages compared to similar products on sale (Figure S10, Supporting Information).

### 2.10 Potential Mechanism of Dsilma‐R@Z in Angiogenic Wound Healing

The previous sections of this study showed that our designed R@Z delivery system for slow release of 2dDR promoted vascularization regeneration; moreover, it appears that only nano‐ZIF‐8 loaded hydrogel facilitated angiogenic activity, whereas Dsilma alone did not demonstrate to have a significant angiogenic effect. Therefore, we explored the reason for this phenomenon by further comparing the biological mechanisms of R@Z and ZIF‐8 in Dsilma. Unlike the evaluation of the flat culture system in the scratch experiment, the use of Matrigel to evaluate angiogenesis in a 3D environment could more intuitively reflect the migration and tube‐forming ability of vascular endothelial cells, particularly the ability to remodel the extracellular matrix.^[^ ^41^ ^]^ As shown in Figure 7a,b, the tube formed using HUVECs under Dsilma‐R@Z treatment was significantly longer than those of the control group, and Dsilma‐Z also provided a slightly positive effect. Moreover, the advantages of nano‐ZIF‐8 and R@Z in forming simulated vascular junctions were more prominent (Figure 7c). Considering the differences in the formation of vascular mimics, we further used transcriptome analysis to investigate the possible differences in the roles of Dsilma‐R@Z and Dsilma‐Z. According to Kyoto Encyclopedia of Genes and Genomes (KEGG) analysis, compared with the control group, Dsilma hydrogels loaded with ZIF‐8 could significantly activate the Wnt signaling pathway of HUVECs, whereas Dsilma‐R@Z possessed the added advantage of better activating the VEGF signaling pathway than Dsilma‐Z (Figure 7d).

To determine the specific contribution of ZIF‐8 in the Dsilma‐R@Z hydrogel healing wound, we first observed that the key for ZIF‐8 regulating HUVECs was Zn^2+^ (Figure 7e). Further analysis of the expression levels showed that loading ZIF‐8 did not lead Dsilma to directly upregulated *Vegfa* but activated matrix metalloproteinases such as *Mmp9* in the Wnt signaling pathway, which serves as an important initiating factor in endothelial cell migration^[^ ^42^ ^]^ (Figure 7f). Then, as shown in Figure 7g, the expression levels of a few key nodes in VEGF and Wnt pathways were repeated by additional reverse transcription polymerase chain reaction, which verified that Dsilma‐R@Z activated the above two signaling pathways.

In summary, based on the physiological process of angiogenesis, including the migration of vascular endothelial cells to start and leave blood vessels, proliferation and combined formation of vascular precursors, and finally constituting mature blood vessels, we summarized the synergistic behavior of Dsilma‐R@Z in promoting angiogenesis. First, R@Z was slowly released from Dsilma, and then, Zn^2+^ in R@Z structure was delivered to activate the Wnt pathway of vascular endothelial cells, and increasing the expression of MMP accelerated vascular endothelial cell migration in a 3D environment. Further, 2dDR was also delivered to tissues by the R@Z drug‐loading system to activate the VEGF pathway, followed by assembling the outlier vascular endothelial cells mobilized by Zn^2+^, accelerating cell proliferation, thus ultimately promoting the formation of new blood vessels (Figure 7h).

## 3 Conclusion

In this study, a strategy using the MOF system as a bridge for multiple modifications is used to break through the limitations of SF hydrogel in the field of skin tissue engineering, thus building a reliable Dsilma‐R@Z hydrogel for advanced wound care. After photocuring and tissue bonding grafting, the ZIF‐8‐based MOF system, R@Z, is chosen to complete structural modification by which overcoming the mechanical weakness of hydrogels relies on the micro‐level cooperation on protein secondary structures. Meanwhile, the antimicrobial and angiogenesis functions of Dsilma‐R@Z are also dominated by the R@Z system, which eliminates the increased risk of bacterial adhesion of gelled silk fibroin as well as enhanced the ability to accelerate tissue healing. Specifically, Dsilma‐R@Z could be injected or sprayed and then cured by light thus providing adaptive protection for wounds with different shapes. Further protection for a wound could rely on the long and short‐acting antibacterial function, which can be built by adjustable ROS‐producing response and Zn^2+^ sustained release. Moreover, the synergistic promotion of angiogenesis from Dsilma‐R@Z could escort the healing of various skin wounds, whether it is a common incision and skin defect, or ischemic trauma.

## 4 Experimental Section

### Materials

Water‐soluble SF was purchased from Yongqinquan Intelligent Equipment Co., Ltd. (China). Zinc nitrate hexahydrate and 2‐methylimidazole (MeIm) were purchased from Aladdin Biochemical Technology Co., Ltd. (China). 2‐Deoxy‐D‐Ribose (2dDR), poly(vinyl alcohol) (Mw 85–124 kDa), glycidyl methacrylate, sodium hydroxide, and 3,4‐dihydroxy‐D‐phenylalanine(DOPA) were purchased from Sigma Aldrich (USA). FITC, TRITC phalloidin, DAPI, and Tris‐buffer were purchased from Beijing Solarbio Science & Technology Co., Ltd. (China).

### Synthesis and Characterization of the R@ZIF‐8 Nanoparticles

R@ZIF‐8 nanoparticles were initially prepared according to previously reported “one‐step method” with a little modification.^[^ ^17b^ ^]^ TEM, SEM, and elemental mapping images were obtained using SEM (FEI Hillsboro, USA) and TEM (Tecnai G2 F20 S‐TWIN), respectively. X‐ray diffractometer (XRD, X'Pert Pro MPD DY129) was used to test the crystal structure within 5°–50° diffraction angle samples. Zeta potential was measured by a Malvern Instruments Zetasizer Nano ZS90. Details on other synthesis and characterizations are presented in the Supporting Information.

### Preparation and Characterization of the Dsilma‐R@Z

Detailed synthetic procedures for Silma and PVA‐DOPA are provided in the Supporting Information. First, 0.05 g LAP photoinitiator was protected from light and dissolved in 20 mL PBS at 45 °C for 15 min. The PVA‐DOPA was dissolved in the LAP photoinitiator solution at 100 °C under the condition of dark and N_2_ protection. After the PVA‐DOPA was completely dissolved, the temperature was lowered to normal temperature for further use. Silma was dissolved in the LAP photoinitiator solution with gentle stirring at room temperature and protected from light. Silma and PVA‐DOPA were mixed in a volume ratio of 3:1, and R(M)@ZIF‐8 (2 mg mL^−1^) was added and mixed evenly to prepare Dsilma‐R@Z. More characterization details of Dsilma‐R@Z could be found in the Supporting Information.

### Computational Details of AAMD Simulations

The details of all of the AAMD simulations can be found in the Supporting Information.

### Antibacterial Activity In Vitro

To investigate the antibacterial activity of the modified SF‐based hydrogel, *S. aureus* (ATCC 29 213) and *E. coli* (ATCC 8739) were used for the tests and cultured separately in fresh liquid LB medium at 37 °C. Control, Dsilma, Dsilma‐Z, Dsilma‐R@Z, and corresponding delay‐time (DT) groups were designed to compare bactericidal activity. The delay groups including Dsilma(DT), Dsilma‐Z(DT) and Dsilma‐R@Z (DT) were a 3‐day delayed immersion solution of related hydrogels and acted in the dark, while other no delay‐time groups were irradiated by simulated sunlight (100 mW cm^−2^) for 2 h, respectively. Live/dead bacteria were imaged using a dual fluorescent dye method (green fluorescent dye [Calcein‐AM] and red fluorescent dye [PI]) under laser scanning confocal microscope (LSCM, FV1200, Olympus). The ROS of *S. aureus*/*E. coli* induced by different samples was detected by DCFH‐DA assay and observed by laser scanning confocal microscope (LSCM). ATP level test was evaluated using an Enhanced ATP Assay Kit (Beyotime). GSH depletion was measured using Ellman's assay. In addition to the previously mentioned 5 groups, 1 mm H_2_O_2_ serving the role of a positive control was added. Each group of sample solution was incubated with Ellman's reagent solution at 25 °C, which was measured after 20 min with a microplate reader at 405 nm. The GSH depletion can be calculated according to the following equation:

(1) $$\mathrm{GSH}\;\mathrm{depletion}=\frac{\mathrm{ODcontrol}-\mathrm{ODsample}}{\mathrm{ODcontrol}}\times 100\%$$

Detailed antibacterial experimental details can be found in the Supporting Information.

### In Vitro Cytotoxicity and Angiogenesis Assay

HUVECs and L929s were seeded into 24‐well plates at a density of 5 × 10^4^, and 1 mL cured Dsilma‐based hydrogels were added into each well. After being cocultured for 1, 3 and 5 days, the relative cell viabilities were determined by a CCK‐8 assay. The early attachment of the cells was investigated on HUVECs and L929s, which were seeded into 24‐well plates containing Dsilma‐based hydrogels at a density of 1 × 10^5^. After 3 h, the culture medium was cleaned, fixed, and then dehydrated by graded ethanol for SEM observation. Similarly, the inspection of cell morphology was following the standard procedures stained with TRITC or FITC phalloidin and DAPI. The safety assessment of the use of blue light during Dsilma‐based hydrogels curing was detected by a ROS kit (R252, Dojindo, Japan) following with flow cytometry (BD Accuri C6). For angiogenesis assay in vitro, scratch test was performed by coculturing HUVECs with Dsilma‐based hydrogels, and the observation time point at 0 and 24 h after scratching. Results of chemotaxis experiments were based on the use of Transwell plates, in which different groups of the hydrogels were cured in a lower chamber as attraction. After being crystal violet stained, acetic acid was used to dissolve color and do quantitative analysis. Detailed cell culture and experimental details can be found in the Supporting Information.

### Hemolytic Activity Assay

The erythrocytes were obtained from the Sprague‐Dawley rats’ blood. After being washed and diluted, 500 µL of the erythrocytes were mixed with 1 mL of the cured Dsilma‐based hydrogels for 1 h. Then after being centrifuged, the absorbance of the supernatants was read using a microplate reader (ThermoFisher Scientific) with Triton X‐100 and PBS serving as a control. More details about hemolytic assay are presented in the Supporting Information.

### In Vivo Therapeutic Assay

The animal experiments in the study were carried out in accordance with the ISO 10993 standard. All procedures for animal experiments followed all ethical guidelines for laboratory animals and were approved by Ethics Committee of West China Hospital of Stomatology, Sichuan University (No. WCHSIRB‐AT‐2021‐281). Three different wound models were built to evaluate the therapeutic ability of Dsilma‐R@Z, including a rat incision model which was infected (Sprague‐Dawley rats), a rat full‐thickness atypical‐circular skin wound‐infected model (Sprague‐Dawley rats), and a rabbit ischemic cartilage exposed to trauma (New Zealand white rabbit). Briefly, the rats were randomly divided into 4 groups: Control, Dsilma, Dsilma‐Z, and Dsilma‐R@Z. After being anesthetized and with hair shaven, an incision or a full‐thickness wound was created on the dorsal surface of each rat, then seeded with *S. aureus* to build infection. Different Dsilma‐based hydrogels or no treatment were captured to bond the cuts or cover those defects following with simulated sun therapy. Equal numbers of rats in each group were sacrificed at Day 3 in the incision model and defect model, followed with other rats of the defect model sacrificed at Day 14 to evaluate the effects of treatment. For constructing rabbit ischemic cartilage exposed trauma, each rabbit was first anesthetized by Zoletil, and the central arteries at the back of the ear were ligated. A total of four cartilage‐exposed skin defects (two per ear) were made on the front of each rabbit's ear with or without Dsilma‐based hydrogels treatment. All the rabbits were sacrificed at Day 21 for further assay. More details on the computations and operations are presented in the Supporting Information.

### Histology Assessment

First, for histology detection, tissue sectioning of 40‐µm thickness was carried out with the help of a microtome (Leica Biosystems). H&E staining was conducted to perform a micromorphological analysis of wound regeneration and major organs in different phases. Processed tissues were fixed with 4% paraformaldehyde for 24 h, and imbedded in paraffin. Later, hematoxylin and eosin‐stained sections of tissues were observed under light microscope and images were taken. Similarly, samples were treated the same as described above for the deparaffinization and re‐dehydration and then stained according to Masson's Trichrome Staining standard instruction, so was it for Sirius Red staining, Gram‐positive bacteria staining and Safranin‐O Fast staining.

### Transcriptome Sequencing and Bioinformatics Analysis

HUVECs were cultured with the Dsilma‐Z, Dsima‐R@Z or pure complete medium for 3 days. Then the total RNA was obtained via the mirVana miRNA Isolation Kit (Ambion, USA). Sample integrity was measured with an Agilent 2100 Bioanalyzer (Agilent Technologies, USA) followed by being entitled to complete the following assays when the RNA integrity number ≥7. The TruSeq Stranded mRNA LTSample Prep Kit (Illumina, USA) was used to generate the transcriptome sequencing library. Illumina sequencing platform (HiSeqTM 2500 or Illumina HiSeq X Ten) was used for transcription sequencing and 150 bp paired‐end reads were generated. The data were used for further analysis, and the read count value was obtained by bowtie2 and eXpress. The R package DEseq2 was used to screen the differentially expressed genes. 2‐fold changes with *p* value < 0.05 were marked as significant. Then, a series of gene functional enrichment analyses such as GO, Kyoto Encyclopedia of Genes and Genomes (KEGG) and heatmap analyses were carried out to determine the major biological attributes.

### RT‐PCR Analysis

The expression levels of some genes in related signal pathways of HUVECs activated by Dsilma‐R@Z were verified by a quantitative real time PCR, including *Vegfa*, *Raf1* and *Mapk12* in VEGF signal pathway as well as *Mmp9*, *Gsk3b* and *Ctnnb1* in Wnt signal pathway. The HUVECs were cocultured with Dsilma‐R@Z or Dsilma‐Z at a cell density of 2 × 10^5^ cells/mL for 3 days. Then the cells were washed and broken for extracting the total RNA, with being reverse‐transcribed into complementary DNA using a PrimeScript RT reagent kit. qRT‐PCR was carried out by employing iTaq universal SYBR Green Supermix (Applied Biosystems, USA) with the gene‐specific primers listed in Table S3, Supporting Information.

### Statistical Analysis

Data are presented as the means ± standard deviations (S.D). Data from experiments were analyzed with Origin 2018 and GraphPad Prism 8. Statistical analysis was performed with SPSS 22.0 statistical software. One‐way analysis of variance (ANOVA) followed by Tukey's post hoc test was used for comparisons among multiple groups (*p < 0.05; **p < 0.01; ***p < 0.001).

## Conflict of Interest

The authors declare no conflict of interest.

## Supporting information

Supporting Information

Supplemental Video 1

Supplemental Video 2

Supplemental Video 3

Supplemental Video 4

Supplemental Video 5

Supplemental Video 6

## Data Availability

The data that support the findings of this study are available in the supplementary material of this article.
