# Supplementary material for: Structural‐Functional Pluralistic Modification of Silk Fibroin via MOF Bridging for Advanced Wound Care
Source: Adv Sci (Weinh). 2022 Oct 28;9(35):2204553. doi: 10.1002/advs.202204553 (PMC9762304; doi:10.1002/advs.202204553)
Supplement: Supplementary file 1 — Supporting Information [file ADVS-9-2204553-s004.pdf]

Structural-Functional Pluralistic Modification of Silk Fibroin via MOF Bridging for Advanced Wound Care

Zhou Zhu[1,3,*], Yanhua Liu[1,*], Junyu Chen[1], Zihan He[1], Pengfei Tan[2], Yong He[3], Xibo Pei[1], Jian Wang[1], Lin Tan[2#], Qianbing Wan[1#]

[1] State Key Laboratory of Oral Diseases, National Clinical Research Center for Oral Diseases, West China Hospital of Stomatology, Chengdu 610041, Sichuan University

[2] College of Biomass Science & Engineering, State Key Laboratory of Polymer Materials Engineering, Sichuan University, Chengdu 610065, China.

[3] State Key Laboratory of Fluid Power and Mechatronic Systems, School of Mechanical Engineering, Zhejiang University, Hangzhou 310027, China.

[*] These authors contributed equally: Zhou Zhu, Yanhua Liu.

Email: tanlinou@scu.edu.cn; champion@scu.edu.cn

Supplementary experimental figures and tables could be found in this part, including the supplementary results of various wound models and Dsilma-R@Z with some commercial wound gels comparison. Details of methods about this research could also be presented in this Supporting Information, such as synthetic route and supplementary characterization of R@Z and Dsilma-R@Z, supplementary results of AAMD and rheology, etc.

Figures

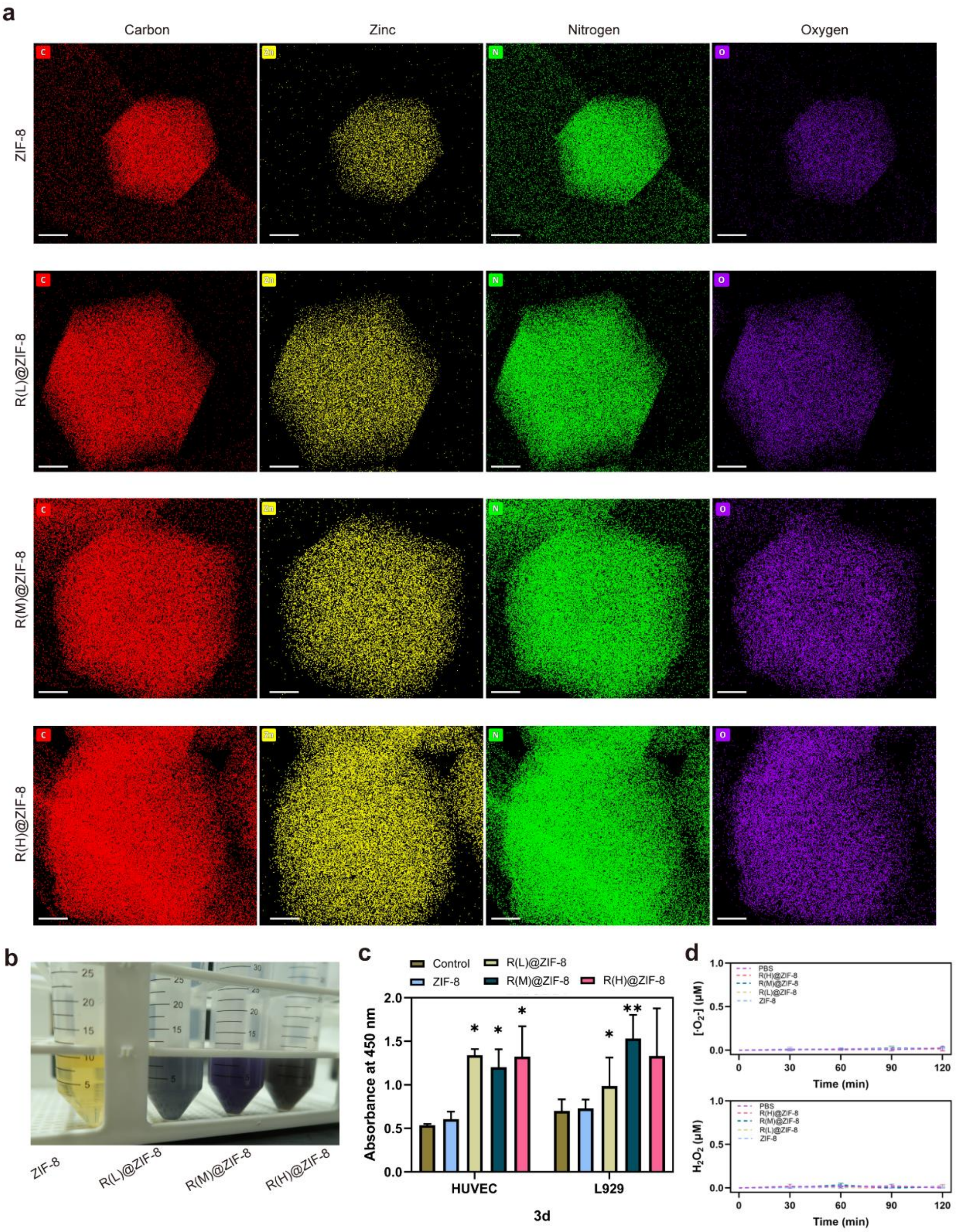

Figure. S1 Characterization of R@ZIF-8. a. TEM mapping images of ZIF-8 and R (L, M, H)@ZIF-8, scale bars: 100 nm. **b.** Color reaction of ZIF-8 and R (L, M, H)@ZIF-8 with Bial's reagent. **c.** CCK-8 assay of HUVEC and L929 after incubated with ZIF-8 and R (L, M, H)@ZIF-8 for 3 days, respectively. n = 3; *P < 0.05; **P = 0.0091. **d.** Superoxide ($\cdot O_2$-) and hydrogen peroxide (H_2O_2) accumulation over time under bule light at 405 nm.

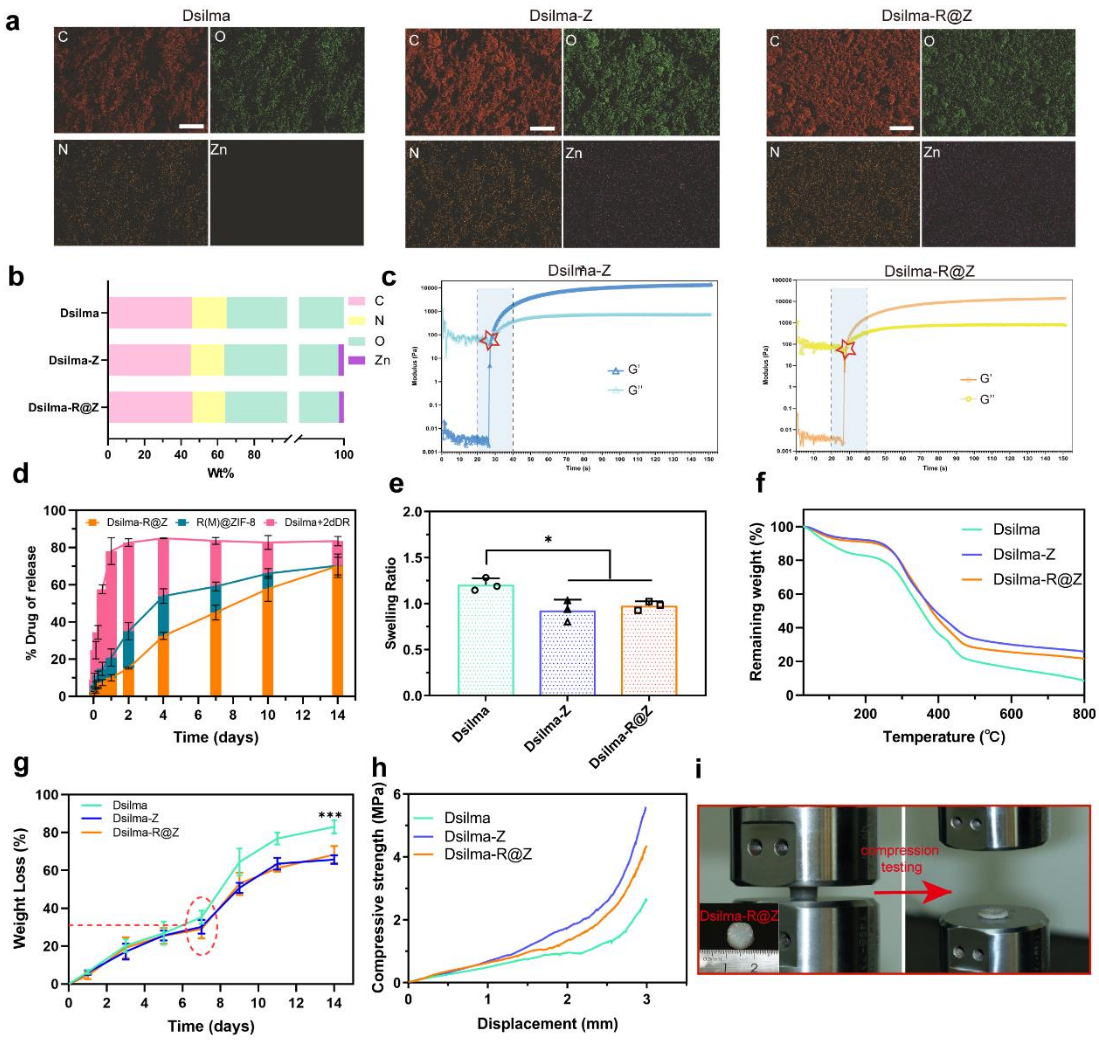

Figure. S2 Synthesis process and characterization of the Dsilma-based hydrogel. a. elemental mapping images of Dsilma, Dsilma-Z, and Dsilma-R@Z. **b.** EDS-based element mass ratio. **c.** The photocuring rheological properties of the hydrogel after MOF adding. **d.** Comparison of 2dDR relative released percent from Dsilma-R@Z, Dsilma+R@Z and single R(M)@ZIF-8. **e.** Swelling ratios of the cured Dsilma-based hydrogels. n = 3; $*P < 0.05$. **f.** TG analysis of the cured Dsilma-based hydrogels. **g.** The degradation of the hydrogels. n = 3; $***P < 0.001$. **h, i.** Compressive loading–unloading curves of the Dsilma-based hydrogels.

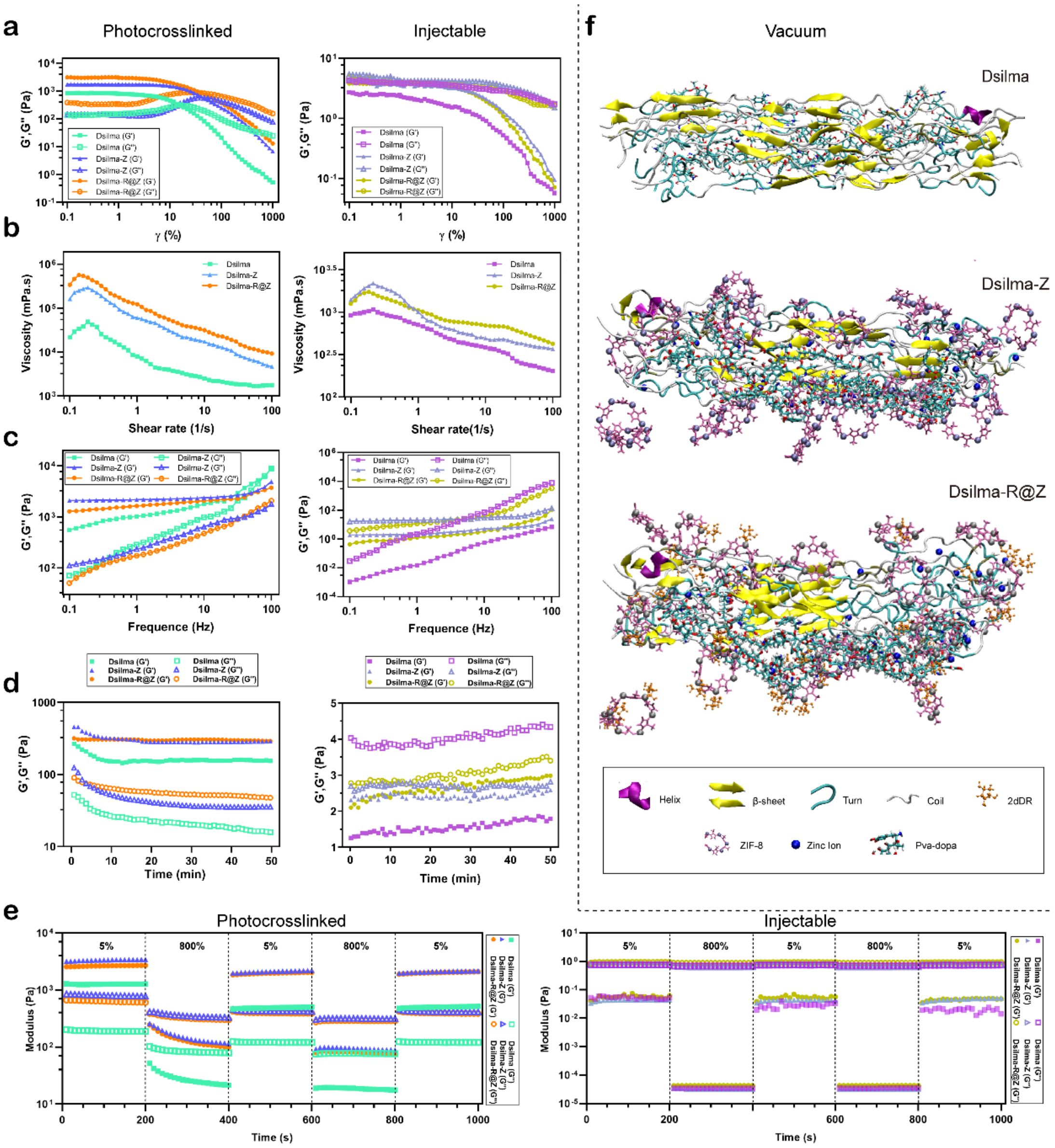

Figure. S3 Rheological properties and molecular simulation. a. Amplitude sweep performed of Dsilma-based hydrogels in photocrosslinked and injectable state with the shear strain increasing from 0.1% to 1000%. **b.** Continuous flow experiments showing the viscosity of the hydrogels in two states plotted against the shear rate. **c.** Frequency sweep performed of Dsilma-based hydrogels in injectable and photocrosslinked state with the frequency increasing from 0.1 Hz to 100 Hz. **d.** Time sweep performed of hydrogels in injectable and photocrosslinked state within 50 min.

e. The resluts of 3itt rheological testing in different states (Strain 5%, 800%). **f.** Representative snapshots of the Dsilma-based hydrogels in vacuum state.

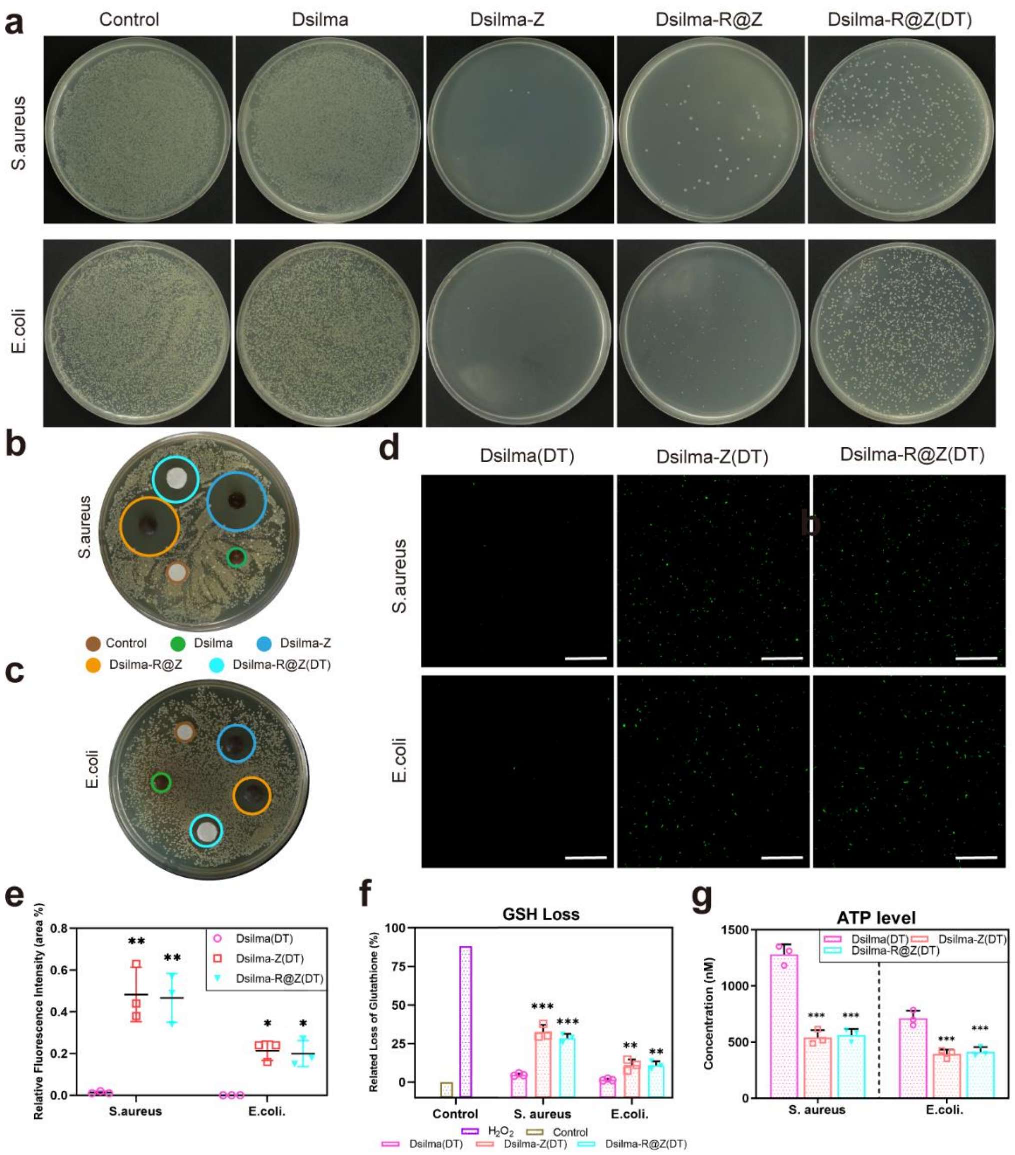

Figure. S4 Detection of bacterial growth inhibition. a. Photographs of the agar plate colonies of *S. aureus* and *E. coli* treated with normal saline, Dsilma, Dsilma-Z, Dsilma-R@Z and Dsilma-R@Z (DT). **b. and c.** The zone of inhibition test. **d.** Fluorescence image of ROS level in S. aureus and E. coli cells after treatment and **e.** Relative ROS levels. n = 3; **P < 0.05, *P < 0.01, ***P < 0.001. Scale bar: 30 μm. **f, g.** Quantitative statistics of ATP level and GSH loss of S. aureus and E. coli cells after different treatments. n = 3; **P < 0.01, ***P < 0.001.

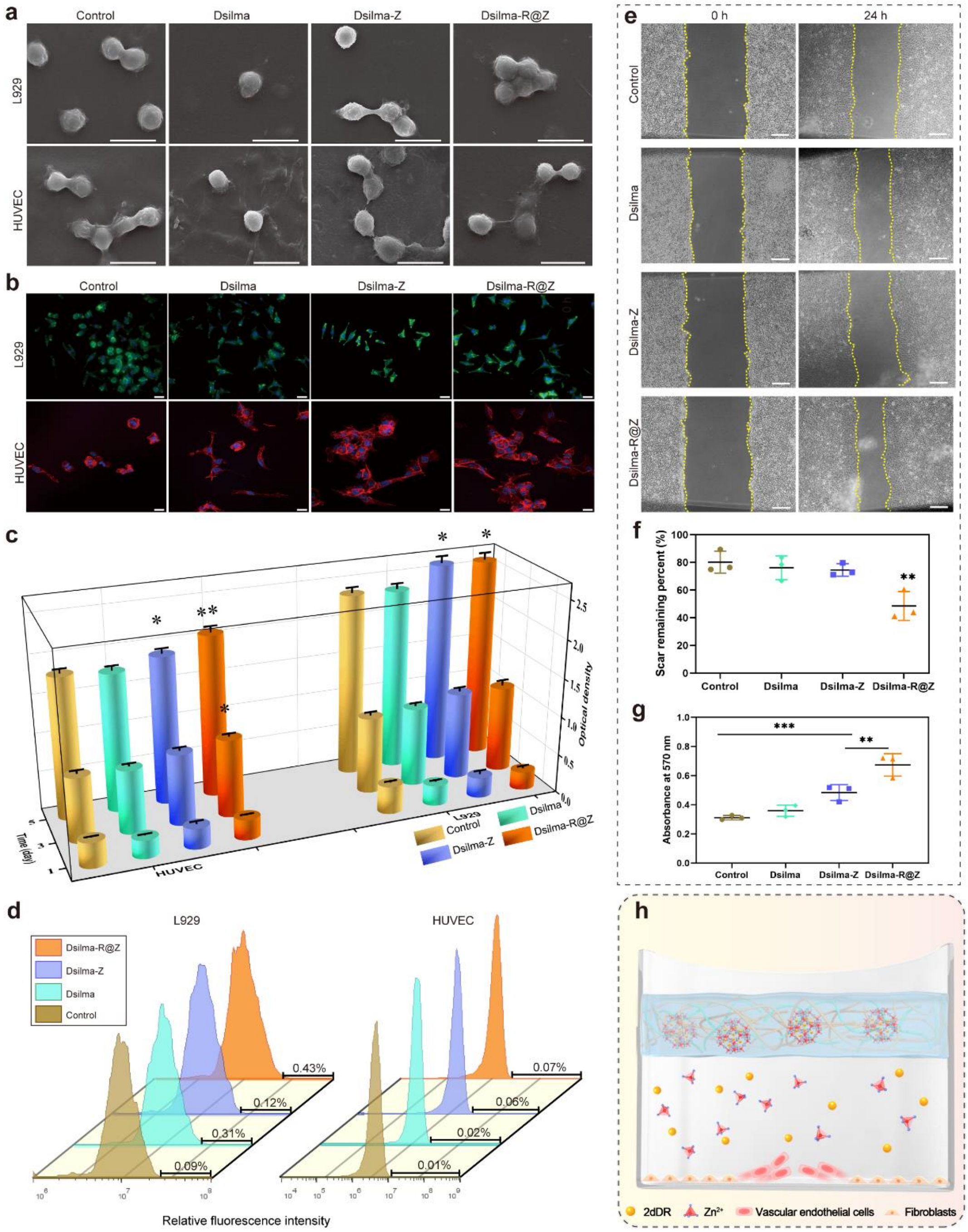

Figure. S5 *In vitro* biocompatibility and angiogenesis evaluation. a. SEM images of HUVECs and L929 cells early adhesion on different hydrogels. Scale bar: 20 μm. **b.** Fluorescence images of the morphology of cells adhered on the hydrogels' surface. Scale bar: 20 μm. **c.** Proliferative viability of HUVECs and L929 co-cultured with Dsilma-based hydrogels for 1, 3, and 5 days. $*P < 0.05$; $**P = 0.0011$, vs. Control group, n = 5 per group. **d.** Flow cytometry results assessed the effect of the photocuring process on intracellular ROS levels. Values indicate the ratio of ROS-positive damaged cells. **e. f.** Migration and quantitative of HUVECs treated with Dsilma-based hydrogels after 8 hours, $**P = 0.0062$, n = 3 per group. Scale bar: 20 μm. **g.** Quantification of crystal violet staining for HUVECs chemotaxis assays. $**P = 0.0083$; $***P < 0.001$, n = 3. **i.** Dsilma-R@Z could safely coexist with those common wound recovery-related

cells and accelerate the migration of vascular endothelial cells.

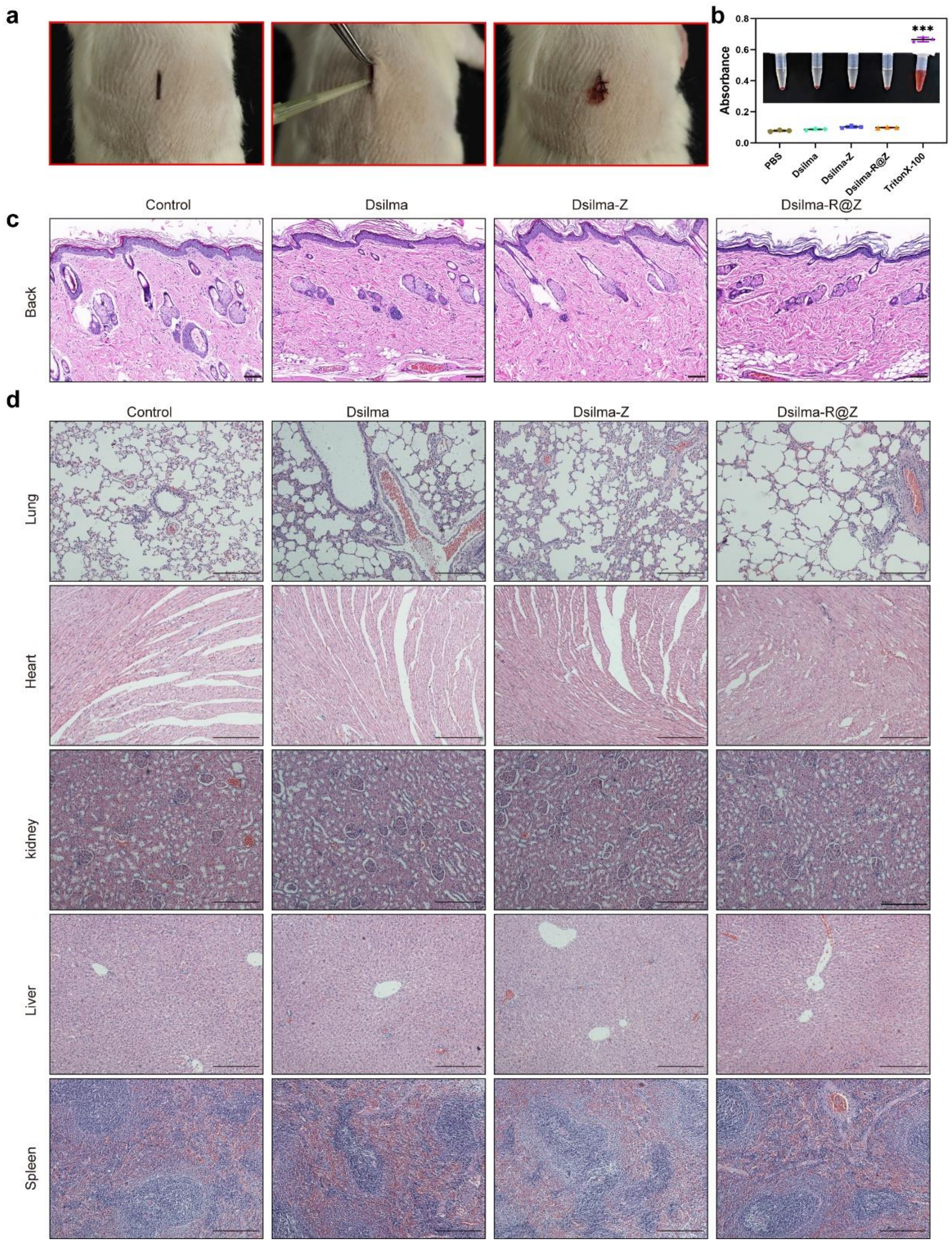

Figure. S6 *In vivo* biocompatibility. a. Surgical demonstration of *in vivo* biocompatibility. **b.** Hemolysis of different hydrogel groups. n = 3, ****P* < 0.001. **c.** Hematoxylin and erosion staining of rat skin tissue after implantation of different Dsilma-based hydrogels after 21 days. **d.** H&E staining of major organs (the heart, liver, spleen, lung, and kidney) after subcutaneous implantation of different hydrogel in the backs of rats for 21 days. All scale bar: 100 μm.

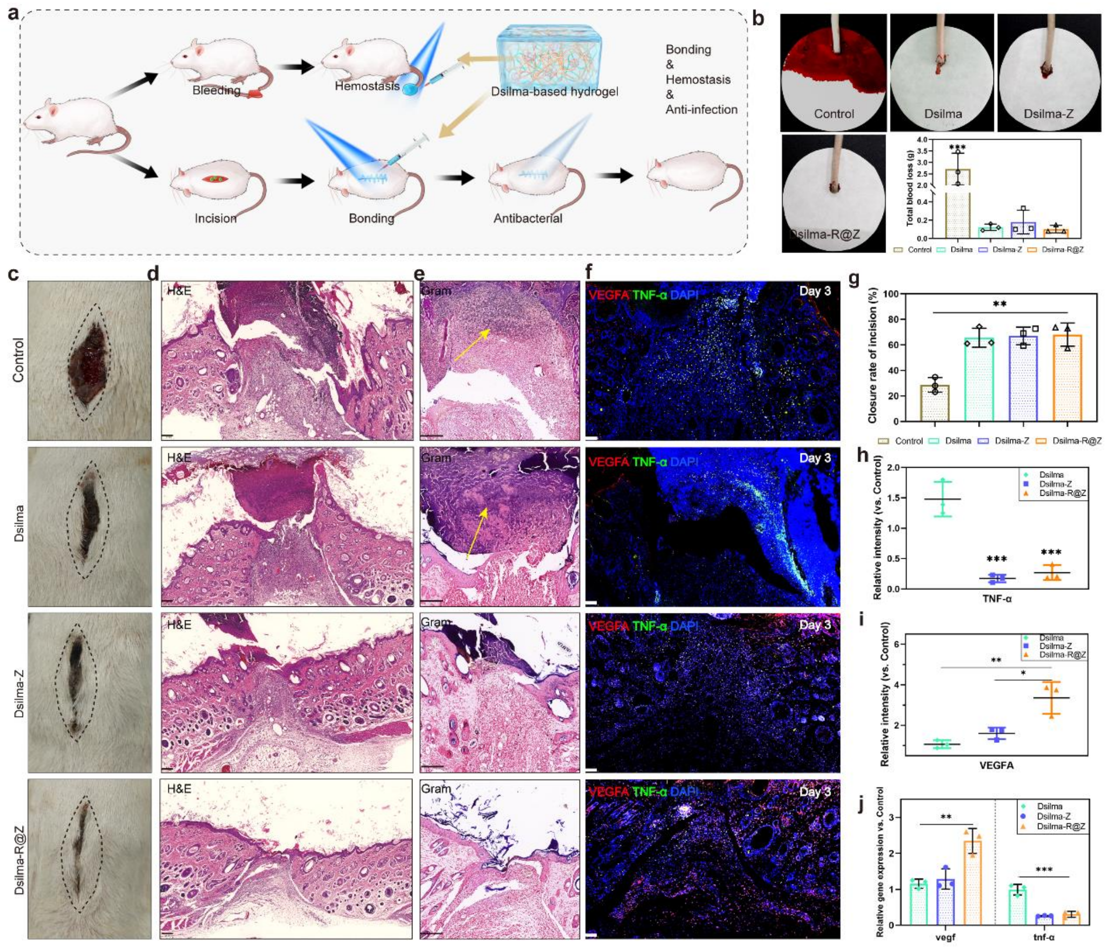

Figure. S7 *In vivo* therapeutic efficacy of conventional incisions. a. Schematic representation of the hemostasis experiment and incision model. **b.** The ability of Dsilma-based hydrogels to treat rat tail bleeding. ***P < 0.001, n = 3 per group. **c.** Digital images of the incisions after 3 days with no hydrogel treatment (control), and under treatment of Dsilma, Dsilma-Z as well as Dsilma-R@Z. The initial incisions' area was marked by dotted lines. **d.** H&E staining of the wounded skin at Day 3. **e.** Gram staining of the incision area at Day 3. **f.** Immunofluorescence staining of vascular endothelial growth factor A (VEGFA, red), alpha tumor necrosis factor (TNF-α, green) and Nuclei (DAPI, blue) for the tissues at Day 3. **g.** The closure rate of incision compared with Control group. **P < 0.01, n = 3. **h, i** Relative VEGFA and TNF-α fluorescence intensity quantification vs Control group. *P = 0.0118; **P = 0.0032; ***P < 0.001. n = 3 per group. All above scale bars equal to 200 μm. **j.** Gene expression levels of *vegf* and *tnf-α* in wound tissue. n=3; **P < 0.01; ***P < 0.001.

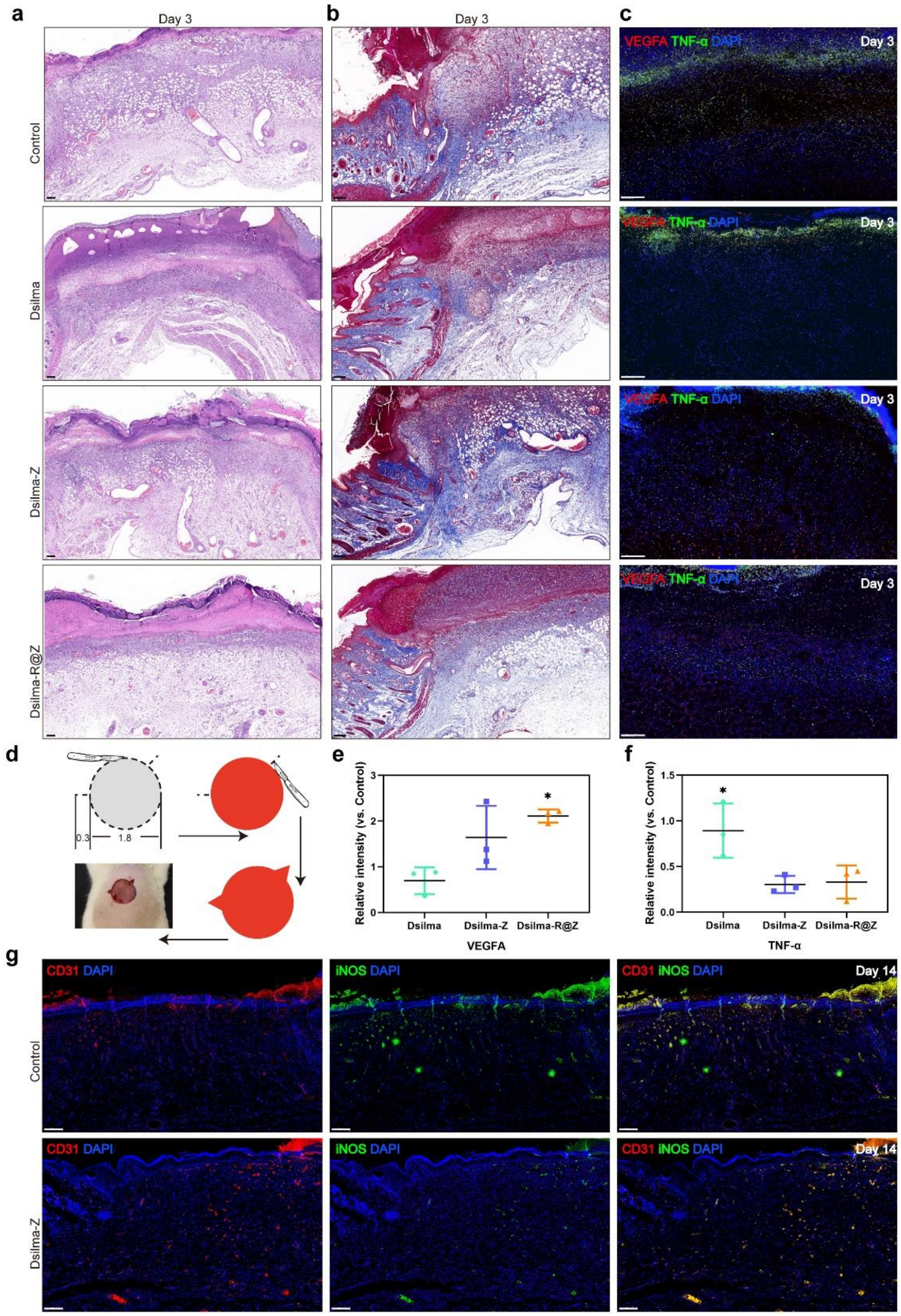

Figure. S8 *In vivo* therapeutic efficacy of atypical-circular skin defects. a. H&E staining of the wounded skin at Day 3. **b.** Masson staining of the wounded skin at Day 3. **c.** Immunofluorescence staining of vascular endothelial growth factor A (VEGFA, red), alpha tumor necrosis factor (TNF-α, green) and Nuclei (DAPI, blue) for the wounds at days 3. **d.** A schematic diagram of surgical pattern for atypical-circular defect. **e.** Relative VEGFA fluorescence intensity quantification vs Control group (*P = 0.0182) and **f.** TNF-α (*P < 0.05), n = 3. **g.** Immunofluorescence staining of Platelet endothelial cell adhesion molecule-1 (CD31, red),

inducible nitric oxide synthase (iNOS, green) and Nuclei (DAPI, blue) for the wounds at Day 14. All above scale bars: 200 μm.

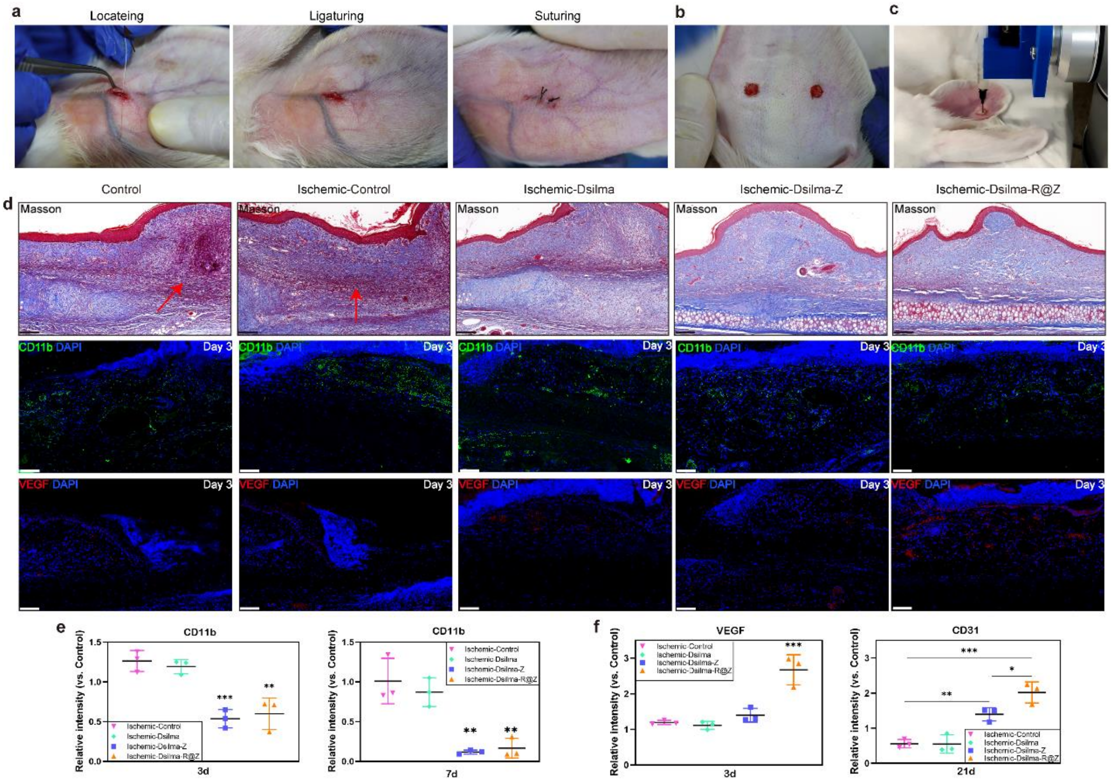

Figure. S9 *In vivo* therapeutic efficacy of Dsilma-R@Z for complex ischemic trauma. a. Surgical demonstration of creating the ischemic microenvironment. **b.** Digital image of initial wound shape. **c.** A surgical robot assisted therapy. **d.** Masson staining (scale bar = 500 μm), immunofluorescence staining of CD11b (green) and Nuclei (DAPI, blue) for wounds at Day 3, immunofluorescence staining of VEGF (red) and Nuclei (DAPI, blue) for the wounds at Day 3, scale bar = 200 μm. Irregular collagens were figured by red arrows. **e.** Relative CD11b fluorescence intensity quantification vs Control group. n=3; $**P < 0.05$, $***P = 0.001$. **f.** Relative VEGF and CD31 fluorescence intensity quantification vs Control group. $*P = 0.0407$; $**P < 0.01$; $***P < 0.001$. n = 3.

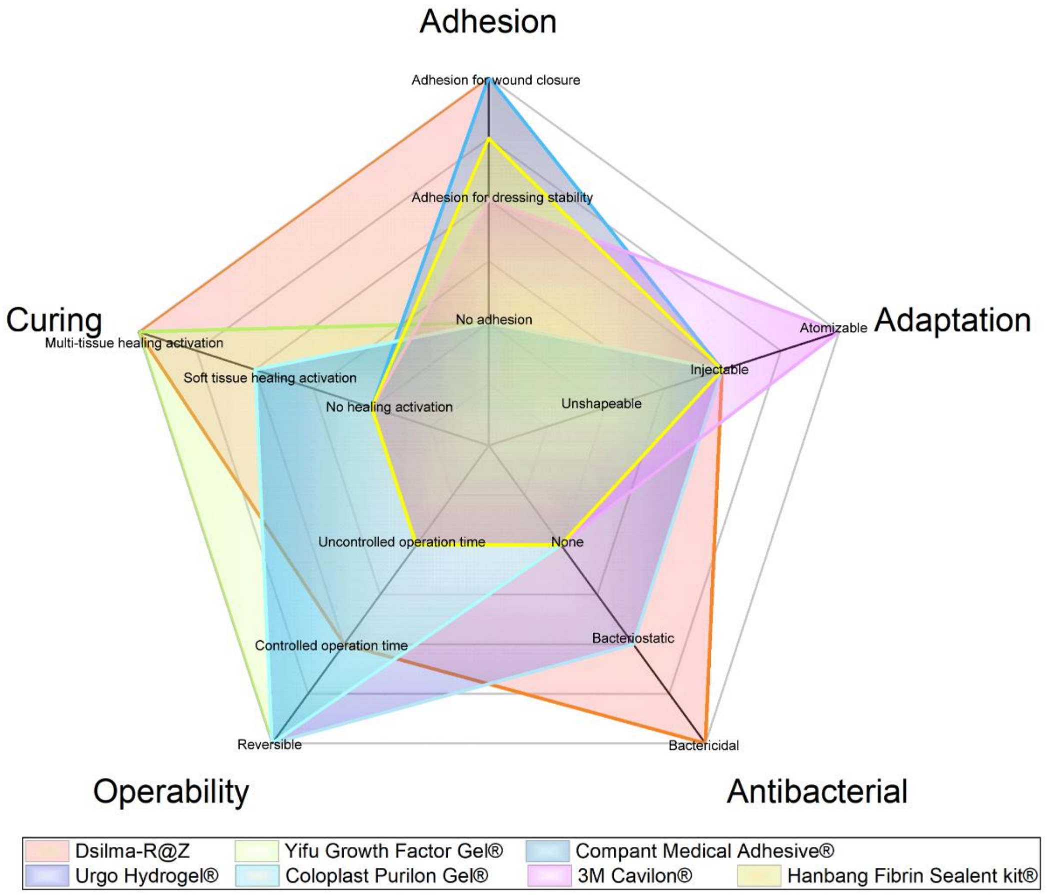

Figure. S10 Application comparison. Comparison of the performance of Dsilma-R@Z hydrogel with some commercially available wound treatment hydrogel.

Tables

Samples	Molar ratio of DOPA / PVA	Degree of substitution of catechol calculated from 1H-NMR (%)
PVA-DOPA1/2	1:2	20
PVA-DOPA3/4	3:4	47
PVA-DOPA4/5	4:5	78
PVA-DOPA1/1	1:1	69

Table. S1 Ratio and corresponding substitution rate of different PVA-DOPA.

Name	DLE	DLC
R(L)@ZIF-8	66.13±2.35%	5.53±0.67%
R(M)@ZIF-8	47.53±2.34%	10.33±1.53%
R(H)@ZIF-8	29.63±2.14%	12.93±1.3%

Table. S2 DLE and DLC of different R@ZIF-8.

Table. S3 Primers for Quantitative Real-Time PCR.

	Name	Primer	Sequence (5’ -3’)
	Vegfa	Forward	CAGAAGGAGGAGGGCAGAAT
	Vegfa	Reverse	TGCGATTGGATGGCAGTAGCA
	Raf1	Forward	GGGAGCTTGGAAGACGATCAG
	Raf1	Reverse	ACACGGATAGTGTTGCTTGTC
	Mapk12	Forward	AGTGGCTTTTACCGCCAGG
	Mapk12	Reverse	GACTGGAAGGGCCGATACAG
human	*Gsk3b*	Forward	GGAGGAACTCACCGACCTTTG
	Gsk3b	Reverse	CGTCCGTAACGCTTCCCAC
	Mmp9	Forward	TGTACCGCTATGGTTACACTCG
	Mmp9	Reverse	GGCAGGGACAGTTGCTTCT
	Ctnnb1	Forward	AAAGCGGCTGTTAGTCACTGG
	Ctnnb1	Reverse	CGAGTCATTGCATACTGTCCAT
	β-actin	Forward	GGACTTCGAGCAAGAGATGG
	β-actin	Reverse	AGCACTGTGTTGGCGTACAG
	Vegf	Reverse	GCGGGATTGCACGGAAACTT
	Vegf	Forward	CTCCGATCGGTTTGCCTCCT
	Tnf-α	Reverse	GCATGATCCGAGATGTGGAACTGG
rat	*Tnf-α*	Forward	CGCCACGAGCAGGAATGAGAAG
	β-actin	Reverse	CATCACTATCGGCAATGAGCGGTTCC
	β-actin	Forward	ACGCAGCTCAGTAACAGTCCGCCTA

Methods

Synthesis and Characterization of the R@ZIF-8 nanoparticles

For synthesizing R@ZIF-8, 0.117g Zinc nitrate hexahydrate was fully dissolved in 40 mL deionized water. Subsequently, low, medium, and high concentrations of 2dDR (2, 5, and 10 mg mL−1) were added, and the mixed solutions were magnetic stirred at 800 rpm for 5 min. Next, 2.27 g 2-MeIm was quickly added to the above solution and continuously stirred for 15 min. The product was washed three times with methanol by 8000 rpm centrifugation, and freeze dried to obtain R (L, M, H) @ZIF-8. ZIF-8 was synthesized in a similar way, except that no 2dDR was added. Encapsulation and Release of 2dDR from R @ZIF-8 were determined by Bial's assay. Bial's reagent (a solution of orcinol, HCl and ferric chloride) was prepared according to the previously reported method[1]. A mixture of 2 ml of the synthetic supernatant with the same amount of Bial's reagent was incubated in a boiling water bath for 20 minutes. The

absorbance was then measured at 630 nm using a UV–VIS spectrophotomete (PerkinElmer Lambda 650, USA) and converted into concentrations using a standard curve of 2dDR to give the remnant drug content. Further, the drug loading capability (DLC) and drug loading encapsulation efficiency (DLE) of 2dDR were calculated according to the reported method[2]:

$$\text{DLC (\%)} = \frac{\text{(total amount of added drug} - \text{amount of remnant drug)}}{\text{(amount of drug loaded NPs)}} \times 100\%$$

$$\text{DLE (\%)} = \frac{\text{(total amount of added drug} - \text{amount of remnant drug)}}{\text{(total amount of added drug)}} \times 100\%$$

50mg of R@ZIF-8 was suspended in 10 mL of deionized water for 1, 3, 6 and 12 h and 1, 2, 4, 7, 10 and 14 days at 37℃ without agitation. The released drug concentration was determined by sampling the supernatant at each time point and following the Bial's assay method described above. The sulfa colorimetry method was used to detect the concentration of $\cdot O_2$- at different time points. Besides, a HPA fluorescence probe was used to measure H_2O_2 presenting ROS concentration as previously reported[3]. Using horseradish peroxidase as a catalyst, a fluorescent phydroxyphenylacetic acid dimer formed by the reaction of H_2O_2 with HPA was analyzed using a fluorescence spectrophotometer. In order to initially explore the biocompatibility of R@ZIF-8, HUVECs and L929s were seeded into 24-well plates at a density of 5×10^4 with equal quality drug-loaded powder addition, respectively. After cocultured for 3 days, the relative cell viabilities were determined by a CCK-8 assay.

Synthesis and characterization of the Silma and PVA-DOPA

First, 40 g of SF was dissolved LiBr solution for 1 h at 70 °C with stirring. Then 6 mL of GMA and 1% NaOH were slowly added to the above solution at the same time, and the reaction was stirred for 6 h in the dark. Finally, the solution was transferred to a dialysis bag ((MWCO: 12-14 kDa, SpectraPor, USA), dialyzed with deionized water at 25 °C for 5 days. After that, the obtained Silma solution was centrifuged to remove impurities solution and then freeze-dried for 2days. The samples were stored at -20 °C for later synthesis. The synthesis of Silma was confirmed by 1H-NMR (Bruker DRX, USA) spectroscopy at 600MHz. The PVA-DOPA polymers were synthesized referring to our previous method [4]. First 6 mmol PVA was dissolve in 30ml DMSO at 100°C, then 0.75g NaHSO4·H2O was added. When the solution temperature was cooled to 80°C, DOPA in different doses (nDOPA: nPVA = 1:2, 3:4, 4:5, 1:1 and 5:4) were immediately added, which were represented by PVA-DOPA1/2, PVA-DOPA3/4, PVA-DOPA4/5, PVA-DOPA1/1 and PVA-DOPA5/4). Subsequently, the reaction system was kept at 80 °C for 24 h under the protection of N2. The product was dialyzed (MWCO: 8000 Da, SpectraPor, USA) in DDW for 3 days, lyophilized and stored under vacuum. The successful synthesis of the PVA-DOPA polymers was verified by means of 1H-NMR spectroscopy and FTIR (Thermo Nicolet, USA).

Characterization of the Dsilma-R@Z

The Dsilma-R@Z were examined by SEM, Small-angle X-ray scattering (SAXS, D8 DISCOVER, Bruker, Germany), FTIR and X-ray photoelectron spectroscopy (XPS,

AXIS Ultra DLD, Kratos, UK) and thermogravimetric analyses (TGA, TGA/DSC2, Mettler Toledo, CH). The concentrations of Zn^{2+} released were detected after collection at various time points by inductively coupled plasma atomic emission spectrometry (ICP-AES, IRIS Advantage, Thermo Scientifc, USA). The release of 2dDR from medical gels was also performed using Bial's assay. Adhesion strength test of the hydrogel was carried out with the help of three models of shear tests, peel tests and pull-off tests using fresh pig skins purchased from the local market. Compression testing was performed by shaping and photocuring the hydrogel into a cylinder of 8mm diameter (5mm height). Both adhesion strength test and compression test were performed with a universal testing machine (Instron 5567, USA) with a loading speed of 5 mm/min. For equilibrium swelling of the Dsilma-R@Z, the photocured hydrogel was weighed (W_0) and placed into PBS at 37 °C, and the swollen hydrogel (W_1) was taken out and weighed after 48 h. The swelling ratio was calculated using the following equation:

$$\text{Equilibrium swelling ratio (\%) swollen dry} = \frac{(W_1 - W_0)}{W_0} \times 100\%$$

Rheological experiments

All the rheological properties of the hydrogel were assessed at 20°C with a Physica MCR 301 rheometer (Anton Paar, Austria). For photocuring scans, blue light (405 nm) with a light intensity of 22 cad was given for 20 s after the start of the test. For the amplitude sweep, the strains were varied from 0.1%-1000% and the frequency was fixed at 1 Hz. Viscosity curves were obtained at shear rates from 0.1 to 100 1/s. The frequency sweeps were performed at a fixed strain of 1% and frequencys in the range of 0.1-100Hz. Time sweeps were carried out under the condition of 1 Hz frequency and 1% strain for 50 min. For 3itt thixotropy experiments, alternate step strain sweep tests were performed at a constant frequency of 10 rad s-1. The oscillatory strain was switched between small strain (γ = 5.0%) and large strain (γ = 800%), ensuring a duration of 200 s for each strain value.

Computational details of AAMD simulations.

All of the all-atom molecular dynamics (AAMD) simulations have been performed in the GROMACS[5] (version 2020.6) simulation package, and the Amber99sb-ildn force field[6] was used to describe the silk fibroin (SF) peptide, each containing 43 amino acid residues[7]. The General Amber force field (GAFF2) was used to describe the polymer and small mocules, which had been well parameterized for most organic molecules[8]. The initial structures had 12 SF extended chains packed into 3 antiparallel layers with inter-chain distance of 1.2 nm. The constant volume canonical ensemble (NVT) AAMD run of 50 ns were performed to equilibrate the peptide structures with constraints on the backbone, and the constraint forces in the long-axis directions were decreased from 4000 kJ/mol to 0, while those in other directions were decreased from 800 kJ/mol to 0 in stepwise manners. Three systems were sequentially simulated based on the equilibrated peptide structure both in vacuum and in water:

i). PVA-DOPA chains were added parallel to the equilibrated peptide and sodium or ions were added to neutralize the partial charges of the peptide.

ii). ZIF-8 ring structures with 96 Zinc ions were randomly added to the system.

iii). 60 2dDR molecules were randomly added to system 2.
In solution systems, the TIP3P water model was used to solvate the above three systems and around 192 chloride ions were added by randomly replacing water molecules to neutralize the charge of Zinc ions. The vacuum systems were run under the NVT ensemble and the solution systems were run under the iso-thermo and iso-baric (NPT) ensemble using the nose-hoover and parrinello-rahman methods at 298 K and 1 atm at for 400 ns, respectively. A cutoff length of 1.2 nm was implemented for the non-bonded interactions, and the Particle Mesh Ewald method[9] with a fourierspacing of 0.1 nm was applied for the long range electrostatic interactions. All covalent bonds with hydrogen atoms were constraint using the LINCS algorithm[10].

For plate colony counts, 100 μL of *S. aureus* and *E. coli* (10^8 CFU mL^{-1}) co-cultured with each group for 12 h were spread onto the agar plates, then the agar plates were incubated at 37°C in the incubator. After 24 h, the number of colonies and the antibacterial rate were calculated. Each sample was carried out in triplicates. For morphological characterization, S.aureus and E.coli (10^8 CFU mL−1) were adhered to coverslips for 2h, and then incubated with each group of treatment factors for 24h. Next, the bacteria on the coverslips were fixed with 2.5% glutaraldehyde for 4 h at 4 °C. Subsequently, the fixed samples were dehydrated with sequential concentration of ethanol solutions of 30%, 45%, 60%, 70%, 80%, 90% and 100% for 15 min followed observing using SEM.

***In vitro* cytotoxicity**

All cells used in this study were provide by National Clinical Research Center for Oral Diseases of China. HUVEC and L929 cells were maintained in dulbecco's modified eagle medium (DMEM; Gibco) and minimum Eagle’s medium (MEM; Gibco) supplemented with 10% fetal bovine serum
(FBS; Gibco) and 1% penicillin and streptomycin (100 IU/ml) at 37 °C under 5% CO_2, respectively.
Scratch test was performed by co-culturing HUVECs with Dsilma-based hydrogels (2×10^6 per well) to 12-well plate. Once cells reached 95 % fusion, the scratch of a straight line was made by a sterile 200 μL pipet tip and the shed cells was washed and removed by PBS. The photographs were recorded using inverted optical microscopy at 0 and 24 h after treatment and analyzed by ImageJ software. To assess whether the blue light at 405 nm wavelength used to cure the Dsilma-based hydrogels would cause damage to cells, the ROS formation was dectect by a ROS Assay Kit (R252, Dojindo, Japan). Following the instructions[11], 2,7-dichlorodihydrofluorescein diacetate (H_2-DCFDA) was used with/without N-acetylcysteine (NAC, 10 mM) as ROS scavenger. Briefly, 10 mM H_2-DCFDA stock solution in DMSO was diluted in culture medium to produce a 5 μM working solution. After 1 h incubation at RT, cells were washed twice with pre-warmed PBS and fixed with 4% paraformaldehyde. The fluorescence count of cells was detected by flow cytometry (BD Accuri® C6).

***In vitro* angiogenesis assay.**

For Transwell chemotaxis assay, 1 mL of Dsilma-based hydrogel was cured in the lower chamber. HUVEC (5×10^4) were seeded in Transwell inserts (Corning incorporated, 5 μm pores). After migration for 24 h, the remaining cells in the upside of Transwell insert were cleaned while the downside cells fixed by 4 % paraformaldehyde. After the stained with 0.1 % crystal violet, a 5 % acetic acid solution was added to dissolve that color for detection of the OD value at 570 nm.

Tube formation simulation

The Tube formation simulation were performed according to the protocol. After co-cultured with for 3 days, those HUVECs in Dsilma-Z and Dsilma-R@Z groups were digested and seeded on Matrigel at a density of 5×10^4 for 8 h of coincubation. Then the tube formation ability of endothelial cells was assessed with a microscope (Leica DMi8, Germany), with tubular structures quantified by Image J 6.0 software.

***In vivo* animal culturing**

Male Sprague-Dawley rats (160 ± 20 g) and New Zealand White Rabbit (1.8 ± 0.3 kg) were provided by Chengdu Dashuo Bio-Technology Co., Ltd. (China). All experiments involving animals were carried out in compliance with the Institutional Animal Care and Use Committee of Sichuan University, Chengdu, China. Rabbits and rats were fed a standard laboratory diet with a 12 h/12 h light/dark cycle under SPF conditions and had at least 1 week of acclimatization before any animal experiment.

***In vivo* toxicity assay**

The *in vivo* toxicity of the Dsilma-based hydrogels was examined using male Sprague Dawley rats. The rats were fasted overnight with free access to water and were anesthetized with 1% pentobarbital sodium. 2 mL of Dsilma, Dsilma-Z and Dsilma-R@Z were implanted in the backs 21 days. The animals were then euthanized and the major organs (the heart, liver, spleen, lung, and kidney) were harvested, embedded in paraffin, sectioned, and stained with H&E for histological analysis.

Hemolytic activity assay

The erythrocytes were obtained by centrifuging (1000 rpm) of the Male Sprague-Dawley rat blood for 10 min. PBS was used to wash the erythrocytes following with diluted to a final concentration of 5% (v/v). Then 500 μL of the erythrocytes were added into a 12-well culture plate covered by Dsilma, Dsilma-Z and Dsilma-R@Z.hydrogel. After placed at 37 ℃ for 1 h, the supernatants were obtained by centrifuging (800 rpm, 15 min), from which 100 µL was transferred into 96-well for absorbance read (at 540 nm). 0.1% Triton X-100 served as the positive control and PBS served as the negative control[12].

Hemostasis performance

The hemostatic performance of Dsilma-based hydrogel was evaluated by employing the rat-tail amputation model (Sprague-Dawley rats, male). Briefly, a rat was anesthetized and its tail was cut to make bleeding. After cutting, the tail of the rat was placed in air for 15 s to ensure normal blood loss. Then the Dsilma, Dsilma-Z and Dsilma-R@Z were placed to stop bleeding. Fifteen minutes later, the weight of the filter paper with absorbed blood and hydrogel sample was measured and compared with a control group (no treatment).

***In vivo* incision model**

Male Sprague-Dawley rats were used to build incision model. Following anesthesia by 1% sodium pentobarbital, rats' hair was shaved and sterilized. An incision of 2.5 cm was created on the dorsal surface of each rat, on which 20 μL of the *S. aureus* suspension with an initial density of 10^7 CFU/mL were added. Afterwards, the wound area was treated by hydrogels 2 hour later to confirm the colonization of bacteria, and then using the same sunlight simulation as the *in vitro* antibacterial experiments for two hours after cured. Incision closure (%) was calculated using the formula below:

$$\text{Incision closure } = \frac{\text{Incision area (Day 0)} - \text{Incision area (Day 3)}}{\text{Incision area (Day 0)}} \times 100\%$$

All results were analyzed by one-way ANOVA test. After 3 days, equal number of rats in each group were sacrificed for further histology and immunohistochemistry.

***In vivo* defect model**

The atypical-circular skin defect model was also built on Male Sprague-Dawley rats' back. Following anesthesia by 1% sodium pentobarbital, hair shaved and sterilized, an irregularly shaped full-thickness skin defect was created on the back of rat. Specifically, this atypical-circular wound based on the circle with a diameter of 1.8 cm, where two 0.3 cm derived incisions were made at the 9 o'clock and half past 1 o'clock directions of the circle, respectively. Then 50 μL of the *S. aureus* suspension with an initial density of 10^7 CFU/mL were added lasting for 2 hours. An equal amount of Dsilma-based hydrogels were applied to the entire wound and cured, followed by 2 hours of sunlight treatment. On day 3 to 14, the wound condition was observed and photographed. Then wound area was measured by tracing the wound boundaries on the photos. Quantification of wound healing:

$$\text{Wound area ratio } = \frac{\text{Area (Day 3,7 or 14)}}{\text{Area (Day 0)}}$$

$$\text{Degree of wound healing } = \frac{\text{Area (Day 0)} - \text{Area (Day 3,7 and 14)}}{\text{Area (Day 0)}} \times 100\%$$

All results were analyzed by one-way ANOVA test. After 3 and 14 days, equal number of rats in each group were sacrificed for further histology and immunohistochemistry.

***In vivo* ischemic wound model**

New Zealand white rabbits were selected to construct such complex wounds. To create a relative ischemic microenvironment, the central artery on the back of the rabbit ear was stripped and ligated with sutures[13]. On the front of the rabbit ear, the full-thickness layers of skin were carefully removed to create two equal-sized circular skin defects of 0.6 cm in diameter to expose the cartilage. The internal cartilage structure should not be damaged during the operation, and the distance between the skin defect and the edge of the ear should be kept more than 1 cm. Then, hydrogel from different Dsilma groups were used to treat these wounds. For each rabbit, the wounds in one ear were covered by Dsilma-R@Z and Dsilma respectively, while the

wound in the other ear were Dsilma-Z and the untreated group. The wound condition was observed and photographed at Day 7, 14, and 21. Wound area refers to the area where dark fibrous tissue was still exposed, while the scar refers to the area where the keratinized layer had not fully recovered[14]. All results were analyzed by one-way ANOVA test. After 21 days, all rabbits were sacrificed for further histology and immunohistochemistry.

Immunohistochemistry

The rats' wound tissue was immunohistochemistry stained with and VEGFA and CD31 to evaluate the blood vessel regeneration as well as TNF-α and iNOS to show the inflammation level. For the immunofluorescence staining of anti-VEGFA and anti-TNF-α, the fixed and frozen regenerated wound tissue collected on 3rd day (incision or defect) were stained with VEGFA and TNF-α antibody at the concentration of 5 μg/mL and (Abcam, China). Meanwhile, the wound tissue samples for other immunofluorescence staining were on 14th day, which later stained with Anti-CD31 and iNOS 6 μg/mL (Abcam, China). Then, FITC-conjugated goat anti-rat IgG was used as the secondary antibody to reveal TNF-α or iNOS expression, with also Rhodamine-conjugated goat anti-rabbit secondary antibody for VEGFA or CD31 fluorescence imaging. Besides, DAPI containing solution was used to stain the nuclei. The rabbits' ear tissue was stained with VEGF and CD31 to show the effect of revascularization, as well as CD11b to evaluate level of inflammation. After routine fixation, dehydration and blocking, those ear tissue were stained with anti-CD31, VEGF or CD11b for reaction for rabbits (Thermo Fisher Scientific, USA). Then Fluor 647-conjugated goat anti-mouse IgG for CD31 and VEGF staining, Fluor 488-conjugated goat anti-mouse for CD11b stanning, and DAPI were carried out to image. All the slides were captured by an inverted fluorescence microscope (Olympus).

Reference

[1] J. Patterson, C. Mura, *J Vis Exp* **2013**, 72, e50225.

[2] X. Cheng, Z. Zhu, Y. Liu, Y. Xue, X. Gao, J. Wang, X. Pei, Q. Wan, *Acs. Biomater. Sci. Eng.* **2020**, *6*, 2186.

[3] P. Li, J. Li, X. Feng, J. Li, Y. Hao, J. Zhang, H. Wang, A. Yin, J. Zhou, X. Ma, B. Wang, *Nat. Commun.* **2019**, *10,* 2177.

[4] S. Hu, X. Pei, L. Duan, Z. Zhu, Y. Liu, J. Chen, T. Chen, P. Ji, Q. Wan, J. Wang, *Nat. Commun.* **2021**, *12*, 1689.

[5] B. Hess, C. Kutzner, D. van der Spoel, E. Lindahl, *J. Chem. Theory Comp.* **2008**, *4*, 435.

[6] K. Lindorff-Larsen, S. Piana, K. Palmo, P. Maragakis, J. L. Klepeis, R. O. Dror, D. E. Shaw, *J. Phy. Chem. B.* **2010**, *78*, 1950.

[7] C.-Z. Zhou, F. Confalonieri, M. Jacquet, R. Perasso, Z.-G. Li, J. Janin, *Proteins.* **2001**, *44*, 119.

[8] a) K. G. Sprenger, V. W. Jaeger, J. Pfaendtner, *J. Phy. Chem. B.* **2015**, *119*, 5882; b) J. Wang, R. M. Wolf, J. W. Caldwell, P. A. Kollman, D. A. Case, *J. Comput. Chem.* **2004**, *25*, 1157.

[9] U. Essmann, L. Perera, M. L. Berkowitz, T. Darden, H. Lee, L. G. Pedersen, *J. Chem.*

Phys. **1995**, *103*, 8577.
[10] B. Hess, H. Bekker, H. J. C. Berendsen, J. G. E. M. Fraaije, *J. Comput. Chem.* **1997**, *18*, 1463.
[11] Y. Guan, H. Niu, Z. T. Liu, Y. Dang, J. Shen, M. Zayed, L. Ma, J. J. Guan, *Sci. Adv.* **2021**, *7*, eabj0153.
[12] J. H. He, M. T. Shi, Y. P. Liang, B. L. Guo, *Chem. Eng. J.* **2020**, *394*, 124888.
[13] A. Grada, J. Mervis, V. Falanga, *J. Invest. Dermatol* **2018**, *138*, 2095.
[14] S. Mascharak, H. E. DesJardins-Park, M. F. Davitt, M. Griffin, M. R. Borrelli, A. L. Moore, K. Chen, B. Duoto, M. Chinta, D. S. Foster, A. H. Shen, M. Januszyk, S. H. Kwon, G. Wernig, D. C. Wan, H. P. Lorenz, G. C. Gurtner, M. T. Longaker, *Sci* **2021**, *372*, 362, eaba2374.
